# Regional Innovation System (RIS) as a means for development: Policies, opportunities and challenges in Ethiopia.

**DOI:** 10.12688/f1000research.159772.1

**Published:** 2025-01-06

**Authors:** Kassahun Sube, Tegegn Belay, Filimon Hando, Ashenafi Bayinesagn

**Affiliations:** 1 Regional and Local Development, Addis Ababa University College of Development Studies, Addis Ababa, Addis Ababa, Ethiopia; 2 Regional and Local Development, Addis Ababa University College of Development Studies, Addis Ababa, Addis Ababa, Ethiopia; 3 Regional and Local Development, Addis Ababa University College of Development Studies, Addis Ababa, Addis Ababa, Ethiopia; 4 Social Work, Addis Ababa University School of Social Work, Addis Ababa, Addis Ababa, Ethiopia

**Keywords:** Science Technology and Innovation (STI), Regional Innovation system (RIS), policies, competitiveness, development, research & development

## Abstract

**Background:**

The successful execution of Science, Technology, and Innovation (STI) policies is crucial for developing a strong Regional Innovation System (RIS) in Ethiopia, which is essential for promoting economic growth and enhancing competitiveness.

**Methods:**

This study utilized a document review approach to critically analyze Ethiopia’s current STI policies and legal frameworks within the context of the Regional Innovation System (RIS).

**Results:**

The findings indicate potential solutions to strengthen Ethiopia’s regional innovation system, including increasing funding for research and development (R&D), fostering better collaboration between universities and industries, and cultivating an entrepreneurial culture. The analysis reveals that Ethiopia possesses significant opportunities to enhance its STI capabilities due to its strategic location, abundant natural resources, and growing governmental and non-governmental support. However, it also highlights critical implementation gaps in STI policies, such as inadequate infrastructure, poor coordination among stakeholders, weak university-industry partnerships, and limited R&D investment.

**Conclusion:**

Ethiopia’s STI policies and legal frameworks are essential for driving economic growth, innovation, and competitiveness by fostering an environment that encourages collaboration and technology transfer. Nevertheless, the country faces challenges in effectively implementing these comprehensive policies due to existing barriers like policy gaps and insufficient institutional support. To strengthen its National Innovation System and promote sustainable economic development, Ethiopia must assess current opportunities, address these obstacles, and explore alternative policies that can enhance its STI capabilities.

## Introduction

The promotion of Science, Technology, and Innovation Policies (STIP) is a prerequisite for enhancing competitiveness and sustainable development ( Andrade et al., 2022; Asheim et al., 2020; Krammer, 2017). This could be achieved by facilitating the development of policies and legal frameworks, the establishment of institutions, and financing initiatives that promote economic growth, employability, entrepreneurship, and improved efficiency ( Ali et al., 2024; Andabayeva et al., 2024). Similarly, a nation’s ability to achieve and maintain sustainable, vibrant economic growth is largely determined by how well its institutions and policies foster the exploration of new knowledge, the progress of technology, and the innovative spirits of its businesses ( Khan, 2022; Basheer, et al., 2022). Policies on Science, Technology, and Innovation policies (STIP) are therefore essential to the advancement of a country and are necessary for it to achieve a dynamic, integrated, and self-sufficient economy ( Singh et al., 2022; Chauhan et al., 2024).

Global, regional, and local policies and legal frameworks have been drafted to bring about economic development and competitiveness among nations ( Ali et al., 2024; Muchie & Ezezew, 2022). For instance, the Sustainable Development Goals (SDG), Agenda 2030 stands for collective action on economic, social, and environmental dimensions in a stable and integrated manner. Similarly, the African Union (AU) adopted a long-term Agenda 2063, “The Africa We Want” to foster inclusive growth and sustainable development at local and regional levels. Both the abovementioned policies recognise Science, Technology, and Innovation (STI) is vital role as universal enablers for achieving aspirations and goals. Within the broader framework of AU Agenda 2063, the African Union (AU) launched the Science, Technology, and Innovation Strategy for Africa 2024 (STISA-2024) in 2014 as the regional framework for accelerating the continent’s transition to a knowledge-based, innovation-led economy. Accordingly, pertinent African organizations were created during this process through the implementation of decisions and legal documents into effect, including the Pan-African Intellectual Property Organization (PAIPO), the African Scientific Research and Innovation Council (ASRIC), and the African Observatory for Science, Technology, and Innovation (AOSTI).

To increase the entire economy’s productivity and competitiveness and facilitate the gradual transition from state to private sector-led growth, the Federal Democratic Republic of Ethiopia (FDRE) Plan and Development Commission developed the Home-Grown Economic Reform (HGER) in 2021. Promoting national innovation, research, and technological capabilities; generating a digital economy and broadening its benefits; enhancing national competitiveness and productivity through the development of technology-based industries; and establishing innovation and technology regulatory systems operating procedures are some of its objectives ( Wazza, 2022).

The Strategic Policy also provides the legal framework for the Ethiopian national innovation system for National Science, Technology, and Mathematics Education, Investment Proclamation, Intellectual Property Law, National Research and Development Strategy, Industrial Parks Development Proclamation, and Technology Incubation and Transfer Policy, Higher Education Proclamation (650/2009) of 2009, the Intellectual Property (IP) Rights System, the National Science, Technology, and Innovation (STI) Policy, and the Professional and Program Mix Policy. Similarly, Higher education institutes are highly required to carry out research, including partnerships with industry, to serve the demands of the development of the nation, and the national legislative and policy frameworks encourage research and innovation that enhances knowledge and technology transfer.

The 1994 education and training policy has been considered to possess the foundation for the creation and execution of various legislative frameworks, programs, and strategies by the Ethiopian government to regulate the operations in Ethiopian higher education institutions with regard to research and development and university-industry linkages ( Kahsay, 2017). The national framework emphasizes technology transfer, university-industry collaboration, and strategies to enhance research and technology transfer in Ethiopian universities, grounded in the Research and Technology Transfer Conceptual and Governance Framework of Ethiopian Higher Education Institutions. The Professional and Program Mix Policy requires a 70:30 undergraduate professional allocation that favors science and technology over the humanities and social sciences ( MoE, 2008); this policy contributes to the expansion of training and research collaborations between universities and industry. In addition, the intellectual property rights system was created in 2003 along with the Ethiopian Intellectual Office ( MoE, 2008). The absence of institutional IP policies in public research and development institutions, government-owned businesses, and higher education institutions, however, is the most significant problem; additionally, the enterprises overlook the need for IP protection ( Kahsay, 2017).

## Methods

The study employed a qualitative approach, specifically document review as the primary methodology to conduct a thorough evaluation of the existing Science, Technology, and Innovation (STI) policies and legal frameworks in Ethiopia within the context of the Regional Innovation System (RIS). The main aim was to analyse how these policies correspond with the RIS framework while identifying gaps and opportunities that could inform future policy. The data collection process consisted of a review of relevant documents, which were categorized into four key areas: government publications, including significant policy documents such as the National Science and Technology Policy (2012) and the Investment Proclamation (2020); reports from international organizations like the United Nations and the World Bank; academic literature that examines STI policies in Ethiopia alongside comparative studies in emerging economies; and regional and global initiatives, including frameworks like the Sustainable Development Goals (SDGs).

Data analysis involved thematic coding of the collected documents, focusing on several critical themes: institutional frameworks, which entailed identifying institutions responsible for implementing STI policies; policy gaps and challenges, which examined discrepancies between intended policies and actual practices; opportunities for innovation within Ethiopia’s National Innovation System (NIS); and a comparative analysis of Ethiopia’s STI policies against those of Kenya and Rwanda. This thematic analysis was conducted enabling a comprehensive exploration of the relationships between STI policies, regional innovation dynamics, and economic outcomes.

To enhance the credibility of the findings, a triangulation approach was employed by comparing data from various sources, including government reports, academic literature, and expert opinions. Additionally, feedback from subject-matter experts was sought to validate interpretations derived from the analysed documents. Despite the thorough nature of this document review, certain limitations were acknowledged, such as potential gaps due to reliance on publicly available documents and the evolving nature of policies. The insights generated from this study aim to deepen understanding of how STI policies impact Ethiopia’s regional innovation system and contribute to sustainable development.

### Science, Technology, and Innovation Policies (STIP) in Emerging Economies

Science, Technology, and Innovation Policies (STIP) have gained more attention in the framework of sustainable and inclusive development, even though it is sometimes thought of as a relatively new concept ( Khan, 2022). The Science Technology and Innovation Policyies (STIP) are also referred as innovation policies, industrial policies, science policies, research and development policies, and technology policies. It encompasses a variety of strategic initiatives aimed at fostering innovation efforts while also addressing broader goals, including the improvement of quality of life, the promotion of sustainability, and the advancement of social integration ( Sagar, 2023). Increased investment in research and development is being made as a result of the national policies’ emphasis on science and technology to promote the quick integration of cutting-edge technologies, strengthen international cooperation, and address grand social issues ( Arimoto, 2024).

Emerging economies, including Brazil, India, China, South Korea, and South Africa (BICSS), illustrate that a country’s competitiveness in the global marketplace is contingent upon its proficiency in executing science, technology, and innovation policies. This involves not only the acquisition and adaptation of technical knowledge but also the enhancement of domestic technological capabilities. These countries can close the technological gap with advanced nations and significantly boost productivity ( Salami & Soltanzadeh, 2012).

An examination of the innovation capabilities across eight Asian nations reveals a positive correlation between income levels and both the inputs and outputs of innovation. Countries classified as high- and upper-middle-income predominantly depend on private sector investments in research and development, whereas those in the lower-middle-income bracket, including Indonesia and Bangladesh, are largely supported by public R&D funding. Japan and South Korea stand out with a greater volume of domestic patent filings, in contrast to Malaysia, Thailand, Indonesia, and Bangladesh, which depend significantly on research and development conducted by foreign enterprises. While China has successfully attained substantial innovation outputs through considerable investments in R&D, India has not progressed beyond a lower-middle-income status, primarily due to its limited R&D spending and dependence on government initiatives ( Park and Kim, 2021).

The Lagos Plan of Action, established in 1980, laid the groundwork for the subsequent Lagos Consolidated Plan of Action, which emerged in the early 2000s. This latter plan underscored the significance of Science, Technology, and Innovation (STI) as essential components for fostering socio-economic advancement across African nations ( Khan, 2022; Yongabo & Göransson, 2020). Key objectives of these initiatives included enhancing research capabilities and reforming the higher education landscape. A notable commitment was made for African countries to allocate 1% of their Gross Domestic Product (GDP) towards research and development ( Khan, 2022). The evolution of innovation and science and technology policies in developing nations is being influenced by the rise of a knowledge-based economy, the forces of globalization, and the involvement of various international organizations, as illustrated by the experiences of the BRIC countries ( Gachie and Govender, 2017). However, STI institutionalization in Africa has not kept pace with other regions; many African countries lack sufficient STI management bodies or policies to advance STI successfully. The National Innovation Systems (NIS) in developing nations, particularly those in sub-Saharan Africa, are frequently perceived as “immature” or “catching up” ( Skorge, 2024; Yongabo & Göransson, 2020; Seifu et al., 2022).

In the African context, Science, Technology, and Innovation Policies (STIP) can be reconceptualized as a mechanism for transforming the continent’s rich history, cultural heritage, and artisanal practices into globally recognized creative industries. This would change the way STI is conceptualized and create an ecosystem similar to Silicon Valley, which creates millions of jobs while balancing “grassroots” and “mission-oriented” innovation policies to boost national innovation capacities, encourage inclusive growth, and combat poverty and inequality ( Khan, 2022; Mavhunga, 2017). Evidence showcases that while R&D significantly drives innovation, which in turn enhances firm productivity, the engagement in R&D and innovation is largely influenced by access to skilled labour and financial resources, highlighting the importance of developing mechanisms and policies to promote knowledge production and bolster innovation initiatives ( Keraga & Araya, 2023).

With South Africa and Egypt contributing the vast majority of contributions, Africa has the weakest research infrastructure of any continent. It also contributes less than 2% of global research publications, struggles with brain drain, and has a very low innovative profile, with 88% of patent activity concentrated in South Africa. Furthermore, Africa falls short of meeting the GERD target of allocating 1% of GDP for research and development ( Khan, 2022; Mavhunga, 2017). In this regard, the latest updates for Ethiopia, Kenya, and Rwanda showed Gross Expenditure on Research and Development (GERD) as follows Kenya (0.8%), Rwanda (0.7%), and Ethiopia (0.3%) ( Ogada et al., 2022). In Africa, significant development entities, including universities, research institutions, financial organizations, educational bodies, and intermediary organizations, are present; however, they often exhibit limited competencies. Furthermore, these entities frequently lack the necessary connections and institutional capacity to effectively participate in innovation strategies that are responsive to market demands ( Seifu et al., 2022).

### Ethiopia, Kenya, and Rwanda Experience

The Global Innovation Index (GII) serves as an annual evaluation that measures the innovation capabilities and achievements of economies across the globe. It includes more than 80 metrics categorized into seven distinct groups: institutions, human capital and research, infrastructure, market sophistication, business sophistication, knowledge and technology outputs, and creative outputs. According to the 2023 Global Innovation Index, each country exhibits distinct characteristics. Ethiopia, categorized as a low-income nation, has a population of 123.4 million, a GDP amounting to 347.8 billion PPP$, and a GDP per capita of 3,434 PPP$, placing it at a rank of 125. In contrast, Kenya, identified as a lower-middle-income country, has a population of 54 million, a GDP of 311.8 billion PPP$, a GDP per capita of 6,122 PPP$, and holds a rank of 100. Similarly, Rwanda, also classified as a lower-income country, has a population of 13.9 million, a GDP of 37.6 billion PPP$, a GDP per capita of 2,836 PPP$, and is ranked 103. This analysis provides a comparative overview of these three nations as reflected in the Global Innovation Index.

The institutional frameworks of Ethiopia, Kenya, and Rwanda show considerable variations that impact their regional innovation systems. Ethiopia’s institutional score of 32.7 is quite low overall, especially regarding the business environment (30.5) and institutional environment (18.6), which limit Ethiopia’s ability to promote innovation. On the other hand, Kenya scored 45.0, indicating some progress toward innovation but still requiring improvements. This is due to a more favourable regulatory environment (49.0) and a moderately supportive corporate climate (30.5). Rwanda is a leader in regional innovation, with a noteworthy overall score of 65.4, driven by a strong business climate (79.1) and a solid regulatory framework (63.2). These differences suggest that Ethiopia and Kenya should learn from Rwanda’s successful policies to strengthen their institutional frameworks and improve their business environments to increase their capacity for innovation. A thorough grasp of the institutional environment is essential for policymakers hoping to boost local innovation systems and stimulate economic growth. In terms of the institutional framework, Singapore, Switzerland, and Finland are the top-ranking countries globally, with scores of 98.4, 87.3, and 85.4, respectively.

Rwanda leads both nations in terms of human capital and research, scoring 22.6, thanks to significant investments in education (37.7) and higher education (26.6), as well as an emphasis on R&D (3.5). Ethiopia may need to strengthen its tertiary education and research initiatives to maximize its innovation potential and competitiveness within the region. This analysis highlights the decisive role that education and research play in nurturing innovation. Kenya and Rwanda are making progress in developing their human capital and R&D frameworks. The disparities in these nations’ approaches to R&D and education show how different investment and emphasis levels can influence their entire innovation ecosystems, underscoring the need for deliberate improvements in human capital development to spur regional advancement. Sweden, Singapore, and South Korea have the highest human capital scores in the world, at 66.9, 63.2, and 62.7, respectively.

The analysis of the infrastructural landscapes in Ethiopia, Kenya, and Rwanda reveals notable differences that have important implications for their different regional innovation systems. Ethiopia has a 12.1% total infrastructure score, indicating that there is room for growth, especially in the Information and Communication Technologies (ICT) category, where Ethiopia scored 17.4. Kenya, on the other hand, has a more solid infrastructure foundation, obtaining a respectable 25.3 and a remarkable 56.4 in ICTs, highlighting the country’s sophisticated digital ecosystem and the government’s dedication to online services and e-participation. With an infrastructure score of 27.9, which emphasizes its effective use of ICTs at 53.7, and a general infrastructure score of 18.3, which reflects its strategic investments in technology and development, Rwanda leads the pack in this comparative assessment. The implications for regional innovation systems are significant: Kenya and Rwanda, with their stronger infrastructure, are better positioned to lead in regional innovation, allowing for increased connectivity, sustainable ecological practices, and greater entrepreneurial activity, while Ethiopia, with its relatively underdeveloped infrastructure, may find it difficult to foster innovation. On the global stage, Finland, Sweden, and Denmark are the leading three states in terms of infrastructure, with scores of 69.2, 67.6, and 65.6, respectively.

When comparing the market sophistication of Rwanda, Kenya, and Ethiopia, certain interesting differences surface that have important ramifications for their respective regional innovation systems. Ethiopia has a 19.8 market sophistication score, compared to Rwanda’s 18.6 and Kenya’s higher 22.1 scores. To be more precise, Kenya scores 21.5, which indicates that it has a strong investment performance and offers a conducive atmosphere for funding innovation. Ethiopia’s investment score, however, lower at 0.4, emphasizes difficulties in bringing in both foreign and domestic funding, which may impede the development of creative businesses. Kenya is probably positioned to maximize its potential for regional competitiveness, although differences in investment and general market sophistication could result in diverse innovation paths. On a global scale, the leading countries in market sophistication are the USA, Hong Kong, and the United Kingdom, with scores of 82.9, 71.8, and 69.3, respectively.

Ethiopia’s business sophistication score of 14.7 indicates difficulties in creating a strong innovation environment. Nonetheless, with a score of 24.2, Kenya tops the list, demonstrating a higher ability to integrate knowledge workers and create innovative connections—two factors that are essential for promoting innovation and technological growth. Rwanda’s business sophistication score of 20.0 indicates a well-rounded strategy, bolstered by its focus on knowledge absorption (26.7) and innovation connections (24.9). Kenya appears to have a more dynamic environment for promoting innovation, which can propel economic development more successfully than Ethiopia’s existing situation, based on its better ratings for knowledge workers (22.7) and innovation links (23.2). On a global scale, Sweden, the USA, and Singapore lead in business sophistication, with impressive scores of 78.5, 69.9, and 65.6, respectively.

The comparative analysis of the knowledge and technology outputs among Ethiopia, Kenya, and Rwanda reveals distinct. Ethiopia’s knowledge and technology outputs stand at 17.9, with a particularly strong emphasis on knowledge creation (19.2) and knowledge impact (24.1). In contrast, Kenya demonstrates slightly higher overall outputs at 18.4, with significantly higher levels of knowledge diffusion (20.4) compared to Ethiopia and Rwanda. Rwanda, with the lowest overall score of 13.6, exhibits strengths in knowledge impact (27.7) but struggles in knowledge creation (8.2) and diffusion (5.1). These differences indicate that while Kenya and Ethiopia are focusing on leveraging knowledge creation and impact to drive innovation, Rwanda’s approach seems to centre on maximizing the impact of existing knowledge. The varied performance in these metrics suggests that fostering regional collaboration and targeted investments in knowledge diffusion and creation could enhance the innovation ecosystems of all three nations, ultimately leading to a more integrated and competitive regional economy. In terms of knowledge and technology outputs, Switzerland, the USA, and Sweden rank as the top three, with scores of 65.3, 63.9, and 63.4, respectively.

A comparative examination of the creative outputs and innovation capabilities in Ethiopia, Kenya, and Rwanda reveals notable differences that have significant implications for their regional innovation systems. As the data indicates, Ethiopia exhibits a creative output score of 4.5, driven predominantly by intangible assets (2.1) and a nascent sector for online creativity (13.6). In contrast, Kenya’s creative outputs are markedly higher at 14.1, buoyed by substantial intangible assets (18.9) and a thriving online creativity score of 17.2. While lower in overall creative output at 6.9, Rwanda shows strengths in intangible assets (7.0) and online creativity (12.2) that reflect its commitment to fostering a digital economy. These disparities highlight Kenya’s more advanced creative economy, emphasizing the need for Ethiopia and Rwanda to enhance their intangible assets and digital creativity initiatives to foster innovation. The implications for the regional innovation system are profound; enhanced collaboration among these nations could facilitate knowledge sharing and resource allocation, ultimately driving a more robust and integrated creative economy across the East African region. Switzerland, the United Kingdom, and Hong Kong lead the global GII in terms of creative outputs and innovative capabilities, with scores of 68.5, 60.0, and 59.2, respectively.

Ethiopia, Kenya, and Rwanda have established legal institutions to coordinate STI activities, with the Ministry of Science and Technology in Ethiopia, the National Council of Science and Technology in Kenya and Rwanda, and the Kenya National Innovation Agency in Kenya. The human resource capacity varies across countries, with Ethiopia and Kenya having a majority of personnel in agriculture and veterinary sciences, while Rwanda has a majority in natural sciences. The majority of personnel are from government institutions in Ethiopia (70.1%) and Rwanda (56.1%), while in Kenya, they are from higher education institutions (56.7%). Rwanda has made progress in STEM education, with 22.4% student enrollment, but female students make up less than a third of this total. The countries have made progress in establishing centres of excellence under the Africa Centre of Excellence program and industrial parks, with Ethiopia leading the region with five industrial parks. There is a pressing necessity to strengthen collaborations between countries, enhance infrastructure, encourage investments from the private sector, and cultivate human resources, particularly emphasizing STEM fields and the participation of women, in order to improve the execution of science, technology, and innovation (STI) initiatives.

### Policy and legal frameworks in Ethiopia

The National Science and Technology Policy, established in 2012, serves as a foundational framework aimed at promoting research, development, and innovation within the nation. It promotes a conducive ecosystem that will support innovation, research, and technology as engines of economic growth and sustainable development. It emphasises public-private partnerships in technology development and commercialisation, strengthens the capacity and efficiency of the research and innovation ecosystem, and creates an environment that is conducive to technology transfer and intellectual property protection. According to the STI Policy, research is an essential part of a strategic plan to effectively adopt foreign technologies, modify them for the local context, employ them successfully, and share knowledge through connections between academia and industry ( Mamo, 2015). The results of research endeavours are anticipated to impact the nation’s progress.

The Ethiopian Ministry of Education introduced a STEM Education program in 2016 to improve the national academic framework. STEM aims to upgrade the standard of instruction in these areas and provide students with the tools they need to succeed in the modern workforce. The initiative serves a crucial function in fostering innovation and advancing technological development within the country. The program seeks to educate students in STEM subjects to produce a workforce capable of fostering innovation and advancing the growth of a knowledge-based economy.

The Ethiopian Investment Commission (EIC) enacted the Investment Proclamation (1180/2020), which offers incentives, guarantees, and protection to investors to encourage their investment in various sectors of the Ethiopian economy. The commission serves as a liaison between government agencies and business sectors, offering guidance, support, and encouragement all through the investment process. Concerning the regional innovation system, the Investment Proclamation functions as a tool for promoting innovation and technology transfer by offering incentives for investments in critical areas like technology, R&D, and innovation. Additionally, the proclamation promotes the creation of creative companies and the uptake of novel technologies.

Ethiopia’s Intellectual Property Law (320/2003) gives researchers, inventors, and business owners the legal means to safeguard their intellectual property to promote innovation and technological development. The proclamation guarantees that entrepreneurs and innovators have the sole right to their projects, including inventions, designs, and works, and it provides the processes for registering and upholding intellectual property rights in Ethiopia. Enforcing intellectual property rights guarantees that innovators can profit from their works and stimulates additional creativity and investment in the nation, all of which contribute to the formation of a conducive environment for innovation.

Ethiopia passed a National Research and Development Strategy in 2021 to advance technological development, innovation, and research in several fields. The strategy describes the top priorities for R&D, which include improving the standard of education, encouraging industry-academia partnerships, and developing the research infrastructure. By concentrating on these areas, the ministry hopes to promote national competitiveness, foster sustainable economic growth, and use research and innovation to address societal issues.

The National Research and Development Strategy offers a road map for orchestrating R&D activities with national priorities, encouraging stakeholder partnerships, and optimising resources to foster economic growth and innovation. The strategy promotes for the development of a supportive environment that fosters research and development initiatives, encourages the generation and dissemination of knowledge, and aids in the commercialization of research outcomes by clearly outlining specific objectives and actionable plans. Employing these initiatives, the ministry strives to strengthen Ethiopia’s innovation ecosystem, cultivate a research and innovation culture, and propel sustainable growth within the nation.

Ethiopia enacted the Industrial Parks Development Proclamation (886/2015) to inspire the foundation of industrial parks throughout the nation to foster industrialisation and economic growth. The proclamation lays forth guidelines for designing, building, and operating industrial parks to bring in foreign and domestic capital, encouraging local businesses to integrate into global value chains and create employment opportunities. Beyond and above, the proclamation aspires to promote manufacturing operations, boost exports, and advance Ethiopia’s socio-economic development by offering incentives and infrastructure support to companies working within industrial parks.

Creating a digital strategy for Ethiopia (2021) is crucial to leveraging the capacity of technology to boost the national economy, enhance social services, and generate employment. Developing a digital strategy for Ethiopia involves incorporating the National Innovation System into account and creating a cooperative ecosystem capable of leveraging the advantages enjoyed by the government, industry, universities, and civil society. Ethiopia has the potential to build a dynamic innovation ecosystem that accelerates digital transformation and quickens economic progress through promoting collaborations and knowledge exchange. This strategy entails funding R&D, enabling tech startups, and encouraging an innovative culture that honours entrepreneurship and inventiveness. By aligning digital strategies with national priorities and leveraging existing resources, Ethiopia can position itself as a leader in the digital economy and drive inclusive and sustainable development for all its citizens.

Addis Ababa University established an innovation and incubation policy in 2022 aimed at fostering the development and success of start-ups and small businesses within the technology sector. According to the policy, the incubators offer emerging businesses access to resources like workspace, funding, networking opportunities, and mentorship in a nourishing environment. On the other hand, innovation policies provide structures and principles for advancing entrepreneurship, technology transfer, and research and development. A conducive setting for innovation, economic growth, and improved global competitiveness is possible to establish by countries through the combination of technology incubation and robust innovation policies. Technology incubation and innovation policies are critical in supporting local talent development, university-industry linkage, and technology transfer.

Professional and Program Mix Policy ( MoE, 2008) offers a comprehensive overview of the professional program mix, annual intake, and enrollment growth in Ethiopian public higher education institutions. It outlines thoroughly the 70:30 undergraduate professional balance favouring technology and science over the arts and social sciences. This approach has ramifications for training and research cooperation between universities and industry. This policy’s potential impact on Ethiopia’s national innovation system makes it relevant.

The Higher Education Proclamation (650/2009) serves as the fundamental legislative framework governing the education system at the national level. This legislation outlines the establishment, functioning, and regulatory compliance of higher education institutions within the country. It sets forth standards for curriculum development, accreditation processes, academic autonomy, and the administration of higher education. The primary goals of the Higher Education Proclamation include ensuring the delivery of high-quality education, fostering innovation and research, and cultivating a skilled workforce capable of contributing to the socioeconomic development of the nation. The proclamation mandates universities to identify their main research areas and themes during conversations with key stakeholders, taking into account the nation’s top priorities and the institution’s competitive advantages; university partnership on research schemes with other domestic and foreign institutes, research centres, and businesses is authorised under the proclamation; the proclamation makes it apparent that collaborations with business are important for research and technology transfer.

The implementation of directives concerning research, technology transfer, university-industry collaboration, and community services for higher education institutions in Ethiopia, as outlined in Directive No: Research 01/2019, aims to strengthen the research and innovation capacities of these institutions. It is clearly stated under the preamble as:

“ *It is mandatory to put in a place and coordinate a well-developed system or research, technology transfer, university-industry linkage, and community services to contribute in ensuring sustainable and holistic development across the nation.”* p1

The objective of the directive is to cultivate an environment of innovation and cooperation among higher education institutions, which will ultimately result in the creation of new technologies, products, and services that serve the broader interests of society. By promoting collaboration between researchers and industry partners, as well as encouraging engagement with the community, these initiatives aim to stimulate economic development and improve the region’s competitive edge. Most importantly, the directive encourages universities to allocate grants and seed money to commercialise inventions in their laboratories and academic units.

### Opportunities for Science, Technology, and Innovation (STI) in Ethiopia

Ethiopian National Innovation System (NIS) has the untapped potential to significantly enhance social development, economic growth, and global competitiveness through science, technology, and innovation. Ethiopia can establish itself as a centre for innovation excellence and propel the innovation landscape of the African continent by capitalising on its advantages, tackling its current problems, and encouraging cooperation either inside or outside the country. Ethiopia is poised to assume a pivotal role in shaping the trajectory of science, technology, and innovation within the region by making strategic investments, forming partnerships, and enacting legislation that fosters entrepreneurship and innovation. Ethiopia has made great progress toward establishing a conducive ecosystem that is favorable to innovation employing policies, initiatives, and partnerships that support research, technology transfer, and knowledge exchange. Establishing research institutes, incubation centres, and innovation hubs that act as catalysts for entrepreneurship and innovation has resulted in the development of a solid foundation in science and technology.

Ethiopia’s national development is driven by several key factors. Primarily, the country possesses a wealth of natural resources and is strategically situated in the Horn of Africa, which provides proximity to the Middle East and its lucrative markets. The remarkable economic growth observed over the past two decades underscores Ethiopia’s potential to cultivate a strong economy, with aspirations to achieve lower-middle-income status by 2025. The government has actively pursued the establishment of a regional Innovation System by developing various policies and legal frameworks related to Science, Technology, and Innovation (STI). This commitment is reflected in increased funding for research and development, the strengthening of institutional capacities, the enhancement of academia-industry collaboration, and the implementation of effective STI policies aimed at diversifying and strengthening the economy.

In alignment with its ambition to attain middle-income status by 2025, Ethiopia has prioritized structural transformation, industrialization, and urbanization. This focus has facilitated significant advancements in the country’s energy, transportation, and telecommunications infrastructure. Central to this economic strategy is substantial public investment in infrastructure, which aims to rectify existing deficits and create a conducive environment for subsequent private sector development. Most significantly, the Ethiopian government has been actively building industrial parks to increase the country’s overall economic growth through the transfer of technology, knowledge, and skills, the creation of productive jobs for the populace, and the export of manufactured goods that earn foreign exchange ( Jote, 2020).

Initiatives are also underway to establish linkages between industrial parks and universities across the nation. Most industrial parks have been located adjacent to universities to accommodate large-scale industrialisation projects. For instance, the Hawassa Industrial Park, which was constructed near Hawassa University, is dedicated to significant domestic and foreign apparel and textile firms with an export revenue target of over US$1 billion and 60,000 employees. The industrial parks of Bole Lemi-I and II, along with the Kilinto Industrial Park situated in the suburbs of Addis Ababa, demonstrate the broader network of industrial zones across Ethiopia, which includes locations in Dire Dawa, Mekelle, Adama, Kombocha, Jimma, Bahir Dar, Debre Birhan, and Aysha Dewalle ( Salmi et al., 2017). In summary, these industrial parks have played a crucial role in advancing Ethiopia’s industrial landscape by generating employment opportunities, enhancing government revenue and export levels, diversifying the range of industrial products, and attracting both Foreign Direct Investment and foreign exchange ( Jote, 2020).

### Challenges

Meaningful STI policymaking cannot be developed, carried out, monitored, or modified without an in-depth understanding of the nation’s context, strengths and weaknesses of the nation’s Science, Technology, and Innovation (STI) agents, their interactions, and incentives as well as barriers that they encounter, all of which are extremely country-specific ( Adenle et al., 2023). Despite the progress made in science, technology, and innovation, Ethiopia still faces challenges in scaling up its innovation ecosystem to meet the demands of a rapidly evolving global market ( Liche & Střelcová, 2023; Gashahun, 2020). One of the most critical challenges is failing to effectively translate the policy into practice. Most policies that come from the top down to the bottom have implementation issues. As Agu et al. (2021) the ‘Top-Down’ approach to policy formulation coupled with limited public awareness of the significance of STI has curtailed efforts in effectively implementing the policy; also, the absence of programs that translate STI policy objectives into action contributes to the inefficiencies in the implementation processes.

Meaningful policymaking in Science, Technology, and Innovation (STI) necessitates a comprehensive understanding of the specific context of a nation, including the strengths and weaknesses of its STI stakeholders, their interactions, incentives, and the barriers they face, all of which are uniquely determined by the country’s circumstances ( Adenle et al., 2023). In Ethiopia, despite advancements in science, technology, and innovation, significant obstacles remain in enhancing its innovation ecosystem to align with the demands of a swiftly changing global market. Ineffective translation of policy into actionable practice is a significant challenge, often due to a top-down approach and lack of public awareness, exacerbated by the absence of tangible action initiatives ( Agu & Ndinda, 2021).

Furthermore, there is a huge gap in coordination and collaboration among various stakeholders in the science, technology, and innovation ecosystem by National Innovation actors. Despite the effective partnerships between government agencies, research institutions, academia, and industry are essential to fostering innovation, knowledge exchange, and technology transfer, the Ethiopian Innovation ecosystem is not conducive and well-orchestrated ( Tekle, 2024; Kebebe, 2019). The STI policy aims to facilitate linkages among stakeholders, but in practice, there is immature coordination and collaboration, resulting in a disconnect rather than synergy, despite its commitment ( Agu & Ndinda, 2021).

Robust advancement in research and development is thought to promote science, technology, and innovation while also boosting competitiveness and economic growth. Research leadership and administration (motivation, teamwork, salary, external influence, political burden, lack of clear vision), communication and outsourcing of research (lack of funds, lack of communication, lack of industrial linkage, lack of partnership, lack of trained journalists), and lack of national strategy & policy (lack of information centre, lack of funds, lack of teamwork, lack of national research centre) are some of the concerns regarding research outputs ( Tesfa, 2015). Furthermore, Gelata et al. (2022) examined e-commerce in Ethiopia and noted that the country’s business had considerable hurdles, including a lack of infrastructure, a lack of trust, security risks, and a lack of a regulatory framework. The Ethiopian industry’s barriers include information technology, inadequate personnel training and expertise, a deficiency in IT infrastructure, customer awareness, and behaviour. The absence of well-structured data warehouses, coupled with inadequately designed websites that provide minimal or no information regarding stakeholders, significantly hinders the understanding of advancements within the sector ( Agu & Ndinda, 2021). Outdated laboratory facilities, insufficient equipment, and above all the poor telecommunication networks in Ethiopia contribute to the poor performance.

The lack of financial resources is the most significant barrier to STI development in developing countries, and Ethiopia is no exception. The critical challenges associated with funding have been categorized into two primary issues. First, there is a significant dependence on state funding coupled with inadequate involvement from the private sector, which has severely hindered science, technology, and innovation (STI) initiatives ( Gobena et al., 2021). Second, the lack of various nationally established guidelines and research evaluation frameworks for the distribution of limited financial resources constitutes an additional obstacle, contributing to inefficiencies and the misallocation of resources ( Agu & Ndinda, 2021).

Concerning the University-industry linkage (Partnership), several efforts exist to create a conducive learning environment for actors to engage in partnerships. Partnerships among National Innovation actors, the status of collective learning among actors is very low in Ethiopia is very low, particularly the roles of R&D institute, TVET College, and University Industries Linkage are very low in collaboration ( Gobena et al., 2021; Ayenew & Teklay, 2017; Ayenew, 2015). In spite of the government’s efforts to enhance and maintain a productive linkage between university and industry, the relation remains in its early developmental stage, with a significant number of outstanding tasks yet to be addressed ( Degaga and Senapathy, 2021).

### Policy recommendations

The role that Science, Technology, and Innovation (STI) policies and legal frameworks play in strengthening Regional Innovation Systems (RIS) and enhancing regional competitiveness has become realised progressively in recent years. Science, Technology, and Innovation (STI) policies significantly determine global, regional, and national social and economic development. The latter part of the 20th century marked a significant formal acknowledgment of this role, largely attributable to the growing dependence on knowledge-based innovation. During this period, the connections among diverse participants within the ecosystem—such as governmental bodies, academic institutions, research and development entities, and industrial sectors—have emerged as essential components ( Galvao et al., 2019). Most importantly, establishing a strong and adaptable STI ecosystem is a dynamic process that calls for ongoing partnerships, fine-tuning, and adaptability. Policymakers must therefore be adaptable, eager to absorb knowledge from ecosystem players, and prepared to change course when new insight becomes available, or circumstances warrant it. Agile policy development is essential to fostering a vibrant innovation ecosystem in which companies, academics, and investors may work together productively to advance novel concepts and technology ( Ratanawaraha et al., 2024).

The Ethiopian government acknowledges the significant importance of Science, Technology, and Innovation (STI). It has therefore enacted various STI policies over the years and established institutions, strategies, and programmes across the key priority sectors to provide opportunities to encourage economic growth. Ethiopia spends about 0.51% of its gross domestic product (GDP) on STIs, which is below the African Union’s (AU) recommended target of 1%. Several stakeholders participate in the STI ecosystem in Ethiopia, with the nature of participation ranging from policy development, regulation, and implementation of STI programs, initiatives, plans, and projects. The different stakeholders have different mandates and exert different levels of power and influence across the STI landscape.

Emerging economies depend on a combination of technological advancement and homegrown innovation to enhance their national innovation systems and policy frameworks, thereby maximizing the advantages of global knowledge transfer for science, technology, and innovation collaboration. This is because many of these nations want to achieve economic growth and competitiveness. Industry, academic institutions, and research centres must change to build mutually beneficial partnerships and complement each other’s strengths to achieve the goals of STI cooperation. Collaborations ought to be established in critical domains such as institutional policy and governance frameworks, higher education in science and technology, entrepreneurship, research infrastructure, legal systems, and technology transfer ( Adenle et al., 2023). The adoption of incremental innovations, prioritizing innovation in sectors with significant economic impacts, concentrating on demand-oriented innovation, implementing spatial innovation, and creating a governmental coordination platform for development priorities and collaboration among ecosystem factors are the five main directions proposed by Shkabatur et al. (2022) for innovation and entrepreneurship policy. These strategies aim to enhance economic growth, improve productivity, and generate jobs.

The key to maximising the effectiveness of the national innovation system is to strengthen research by creating substantial and long-term funding sources, safeguarding the research council already in place, endorsing the professional association, and figuring out how to leverage the tremendous amount of knowledge and expertise held by professionals from Ethiopia’s diaspora ( Tesfa, 2015). To promote economic growth and optimise Ethiopia’s economy’s competitiveness, the national innovation actors ought to be motivated to engage in R&D and learning initiatives, whether by creating new ones or modifying existing ones ( Gobena & Kant, 2022)

Policymakers in Ethiopia ought to focus on increasing the number of citable journals, encouraging patent registrations, and promoting technology exports within the context of research and development (R&D) innovation to stimulate economic growth ( Agezew, 2024). Merely increasing research funding may prove insufficient for economic advancement unless it is paired with targeted R&D initiatives that have a direct economic impact. This underscores the necessity for policies that facilitate collaboration among government entities, academic institutions, and the private sector to enhance innovation and technology transfer. A supportive policy framework, the expansion of industries, the enactment of intellectual property rights protection laws, a burgeoning economy, the establishment of science and technology universities, the development of industrial parks, and improvements in infrastructure represent significant opportunities ( Degaga & Senapathy, 2021). A coordinated effort among government bodies, universities, industries, and other relevant stakeholders is essential to address the aforementioned challenges and revitalize university-industry linkages (UIL). Furthermore, it is imperative to leverage the emerging opportunities identified and to strengthen the enabling environment for UIL to thrive with increased vigor.

The effective optimization of Science, Technology, and Innovation (STI) policies within the nation represents a vital component in fostering economic development and enhancing competitiveness. This can be accomplished through the augmentation of productivity, the minimization of costs, and the enhancement of operational efficiency. The implementation of STI policies holds the promise of generating substantial benefits for the economy as a whole. Therefore, and this is very important, Ethiopia needs to make an effort to maximise STI’s potential to accelerate both its economic growth and social well-being. Despite the previously mentioned initiatives to formulate all-encompassing policies on Science, Technology, and Innovation (STI) and to institute a Regional Innovation System (RIS), the current situation suggests a discrepancy in the efficient execution of the policies.

## Conclusion

The policies and legal frameworks governing Science, Technology, and Innovation (STI) are crucial for Ethiopia’s pursuit of economic development, innovation, and enhanced competitiveness. These frameworks foster an environment conducive to collaborative learning, entrepreneurship, research, and the transfer of technology. The effective operation of the Ethiopian National Innovation System relies on a robust policy and legal foundation, which is established by existing policies such as the National Science and Technology Policy, the Strategic Policy for National Science, Technology, and Mathematics Education, the Investment Proclamation, the Intellectual Property Law, the National Research and Development Strategy, the Industrial Parks Development Proclamation, the Digital Strategy for Ethiopia, the Technology Incubation and Transfer Policy, the Professional and Program Mix Policy, and the Higher Education Proclamation. By evaluating current opportunities, addressing existing challenges, and suggesting alternative policies, Ethiopia can enhance its STI policies to bolster a Regional Innovation System that promotes economic growth, technological progress, and sustainable development.

The study investigated existing barriers in the ecosystem; Ethiopia faces challenges in creating comprehensive STI policies and successfully implementing them into practice. It emphasises how important it is to harness the outcomes of innovations within a strong policy framework and how STI capacity-building is necessary for sustainable development. Among the issues that must be resolved are policy gaps and the requirement for more robust institutional assistance. The policies and legal frameworks governing Science, Technology, and Innovation (STI) are crucial for Ethiopia’s pursuit of economic development, innovation, and enhanced competitiveness. These frameworks foster an environment conducive to collaborative learning, entrepreneurship, research, and the transfer of technology. The effective operation of the Ethiopian National Innovation System relies on a robust policy and legal structure, which is established by existing policies such as the National Science and Technology Policy, the Strategic Policy for National Science, Technology, and Mathematics Education, the Investment Proclamation, the Intellectual Property Law, the National Research and Development Strategy, the Industrial Parks Development Proclamation, the Digital Strategy for Ethiopia, the Technology Incubation and Transfer Policy, the Professional and Program Mix Policy, and the Higher Education Proclamation. By evaluating current opportunities, addressing existing challenges, and suggesting alternative policies, Ethiopia can enhance its STI policies to bolster a Regional Innovation System that promotes economic growth, technological progress, and sustainable development.

### Ethics and consent

Ethical approval and consent were not required.

## Data Availability

No data associated with this article. The article does not contain any associated data because it primarily focuses on the evaluation of existing Science, Technology, and Innovation (STI) policies and legal frameworks in Ethiopia rather than presenting original empirical research findings. It serves as a methodological discussion and analysis of the current state of STI in the context of the Regional Innovation System (RIS), emphasizing policy gaps and recommendations for improvement without generating new data. Therefore, no datasets are available for publication as the content centers on policy analysis and strategic insights rather than quantitative or qualitative research outputs. No extended data associated with this article.
