# National Innovation System (NIS) as a Means for Development: Policies, Opportunities, and Challenges in Ethiopia.

**DOI:** 10.12688/f1000research.159772.2

**Published:** 2025-02-18

**Authors:** Kassahun Sube, Tegegn Belay, Filimon Hando, Ashenafi Bayinesagn

**Affiliations:** 1 Regional and Local Development, Addis Ababa University College of Development Studies, Addis Ababa, Addis Ababa, Ethiopia; 2 Regional and Local Development, Addis Ababa University College of Development Studies, Addis Ababa, Addis Ababa, Ethiopia; 3 Regional and Local Development, Addis Ababa University College of Development Studies, Addis Ababa, Addis Ababa, Ethiopia; 4 Social Work, Addis Ababa University School of Social Work, Addis Ababa, Addis Ababa, Ethiopia

**Keywords:** Science Technology and Innovation (STI), Regional Innovation system (RIS), policies, competitiveness, development, research & development

## Abstract

**Introduction:**

A robust National Innovation System (NIS) has become crucial for fostering socioeconomic progress and competitiveness in emerging economies like Ethiopia, and its success relies significantly on successful implementation of science, technology, and innovation (STI) policies.

**Methods:**

Using a document review approach, the study examined at various STI policies and legal frameworks regarding their relation to the National Innovation System (NIS).

**Results:**

The results of the study indicate that the National Innovation System is supported by the current STI policies and legal fremeworks. There is evidence that an enabling environment emerges to strengthen the NIS, which includes enhancing R&D funding, improving university-industry linkage, and promoting an entrepreneurial culture. Similar to this, research shows that Ethiopia has a lot of potential to increase STI’s capacity because of its advantageous location, wealth of natural resources, and expanding support from the public and private sectors. The execution of STI laws and policies is nevertheless hampered by a number of issues, such as poor R&D investment, weak university-industry linkage, poor coordination among stakeholders, and inadequate infrastructure.

**Conclusion:**

Ethiopia’s STI policies and legal frameworks are essential for driving economic growth, innovation, and competitiveness by fostering an environment that encourages collaboration and technology transfer. Nevertheless, the country faces challenges in effectively implementing these comprehensive policies due to existing barriers like policy gaps and insufficient institutional support. To strengthen its National Innovation System and promote sustainable economic development, Ethiopia must assess current opportunities, address these obstacles, and explore alternative policies that can enhance its STI capabilities.

## Introduction

Science, Technology, and Innovation (STI) is vital in ensuring sustainable development and national competitiveness in the knowledge-based economy ( Andrade et al., 2022; Asheim et al., 2020; Krammer, 2017). This is therefore thought to be possible through supporting the creation of STI policies and regulations, institutions, and funding programs that encourage economic expansion, employability, entrepreneurship, and increased productivity ( Ali et al., 2024; Andabayeva et al., 2024). Likewise, the degree to which a country’s institutions and policies encourage the pursuit of new knowledge, technological advancement, and the inventiveness of its enterprises determines its capacity to attain and sustain viable, dynamic economic development ( Khan, 2022; Basheer, et al., 2022). Therefore, policies pertaining to science, technology, and innovation (STI) are critical to a nation’s progress and are required for it to develop a dynamic, integrated, and self-sufficient economy ( Singh et al., 2022; Chauhan et al., 2024).

At every level—global, regional, and local—policy and legal frameworks have been developed to achieve socioeconomic progress and prosperity ( Ali et al., 2024; Muchie & Ezezew, 2022). For instance, the Sustainable Development Goals (SDG), Agenda 2030 stands for collective action on economic, social, and environmental dimensions in a stable and integrated manner. Similarly, the African Union (AU) adopted a long-term Agenda 2063, “The Africa We Want” to foster inclusive growth and sustainable development at local and regional levels. Both the abovementioned policies recognise Science, Technology, and Innovation (STI) is vital role as universal enablers for achieving aspirations and goals. Within the broader framework of AU Agenda 2063, the African Union (AU) launched the Science, Technology, and Innovation Strategy for Africa 2024 (STISA-2024) in 2014 as the regional framework for accelerating the continent’s transition to a knowledge-based, innovation-led economy. Accordingly, pertinent African organizations were created during this process through the implementation of decisions and legal documents into effect, including the Pan-African Intellectual Property Organization (PAIPO), the African Scientific Research and Innovation Council (ASRIC), and the African Observatory for Science, Technology, and Innovation (AOSTI).

To increase the entire economy’s productivity and competitiveness and facilitate the gradual transition from state to private sector-led growth, the Federal Democratic Republic of Ethiopia (FDRE) Plan and Development Commission developed the Home-Grown Economic Reform (HGER) in 2021. Promoting national innovation, research, and technological capabilities; generating a digital economy and broadening its benefits; enhancing national competitiveness and productivity through the development of technology-based industries; and establishing innovation and technology regulatory systems operating procedures are some of its objectives ( Wazza, 2022).

The Strategic Policy also provides the legal framework for the Ethiopian national innovation system for National Science, Technology, and Mathematics Education, Investment Proclamation, Intellectual Property Law, National Research and Development Strategy, Industrial Parks Development Proclamation, and Technology Incubation and Transfer Policy, Higher Education Proclamation (650/2009) of 2009, the Intellectual Property (IP) Rights System, the National Science, Technology, and Innovation (STI) Policy, and the Professional and Program Mix Policy. Similarly, Higher education institutes are highly required to carry out research, including partnerships with industry, to serve the demands of the development of the nation, and the national legislative and policy frameworks encourage research and innovation that enhances knowledge and technology transfer.

The 1994 education and training policy has been considered to possess the foundation for the creation and execution of various legislative frameworks, programs, and strategies by the Ethiopian government to regulate the operations in Ethiopian higher education institutions with regard to research and development and university-industry linkages ( Kahsay, 2017). The national framework emphasizes technology transfer, university-industry collaboration, and strategies to enhance research and technology transfer in Ethiopian universities, grounded in the Research and Technology Transfer Conceptual and Governance Framework of Ethiopian Higher Education Institutions. The Professional and Program Mix Policy requires a 70:30 undergraduate professional allocation that favors science and technology over the humanities and social sciences ( MoE, 2008); this policy contributes to the expansion of training and research collaborations between universities and industry. In addition, the intellectual property rights system was created in 2003 along with the Ethiopian Intellectual Office ( MoE, 2008). The absence of institutional IP policies in public research and development institutions, government-owned businesses, and higher education institutions, however, is the most significant problem; additionally, the enterprises overlook the need for IP protection ( Kahsay, 2017). Lastly, the study was conducted out primarily to deal with the objectives that follow.

1. To review the STI policies and legal frameworks supporting National Innovation Systems (NIS) in Ethiopia.
2. To explore the opportunities for leveraging National Innovation Systems (NIS) for economic development.
3. To identify the main challenges hindering National Innovation Systems (NIS) from contributing to economic development.

### National Innovation System (NIS)

These days, Science, Technology, and Innovation (STI) policies are now essential to sustainable socioeconomic development as well as national competitiveness in knowledge-based economies (Xu et al., 2022; Surana et al., 2020). Accordingly, promoting SPI policies is a prerequisite to achieving innovation-driven development (Zhu et al., 2022); since STI may improve worker efficiency and improve the calibre of production factors (Zhou et al., 2022). Also, maintaining healthy and sustainable economic development is largely dependent on STI (Walsh et al., 2020). The STI capability is a key indicator of overall competitiveness and a determining element in gaining a competitive edge globally. According to Zhou et al. (2022), Science, Technology, and Innovation (STI) policy is a crucial element of national innovation systems, a vital tool for influencing the innovation environment and promoting innovation vitality, and a basic assurance for innovation-driven development.

The main concern of policy makers in developed and developing countries today is national competitiveness and how competitiveness can be improved (Khyareh et al., 2022). The need to resolve policy failures has led to the development of innovative systems that may refer to the innovation characteristics of either a country or Regional level (Chaparro-Banegas et al., 2023). Innovation systems, which develop continuously over time, are created through collaboration and cooperation among entities within a region or country (Asheim et al., 2011; Doloreux & Parto, 2005). The concept of the National Innovation System (NIS) originated between the end of the 1980s and the middle of the 1990s; Bengt-Åke Lundvall was the first scholar coin the term National Innovation System (NIS) (Lopez-Rubio et al., 2021). It has been in use for over two decades and is now extensively used by researchers and policymakers worldwide (Kurpayanidi, 2021). The significance of systemic collaboration in innovation processes has been emphasized by the NIS concept (Samara et al., 2012). The National Innovation System (NIS), which focuses on a specific territory, is the inspiration for the “Regional Innovation System” (RIS) concept (Iammarino, 2005).

### Science, Technology, and Innovation Policies (STIP) in Emerging Economies

Although widely considered as an emerging concept, Science, Technology, and Innovation (STI) Policies have garnered more attention in the context of sustainable and inclusive development ( Khan, 2022). Research and development policies, innovation policies, industrial policies, scientific policies, and technology policies are other names for the scientific Technology and Innovation (STI) policies. It includes a range of strategic activities that are intended to support innovation efforts while simultaneously addressing more general objectives, such as enhancing social integration, promoting sustainability, and improving quality of life ( Sagar, 2023). Increased investment in research and development is being made as a result of the national policies’ emphasis on science and technology to promote the quick integration of cutting-edge technologies, strengthen international cooperation, and address grand social issues ( Arimoto, 2024).

Emerging economies, including Brazil, India, China, South Korea, and South Africa (BICSS), illustrate that a country’s competitiveness in the global marketplace is contingent upon its proficiency in executing science, technology, and innovation policies. This involves not only the acquisition and adaptation of technical knowledge but also the enhancement of domestic technological capabilities. These countries can close the technological gap with advanced nations and significantly boost productivity ( Salami & Soltanzadeh, 2012).

An examination of the innovation capabilities across eight Asian nations reveals a positive correlation between income levels and both the inputs and outputs of innovation. Countries classified as high- and upper-middle-income predominantly depend on private sector investments in research and development, whereas those in the lower-middle-income bracket, including Indonesia and Bangladesh, are largely supported by public R&D funding. Japan and South Korea stand out with a greater volume of domestic patent filings, in contrast to Malaysia, Thailand, Indonesia, and Bangladesh, which depend significantly on research and development conducted by foreign enterprises. While China has successfully attained substantial innovation outputs through considerable investments in R&D, India has not progressed beyond a lower-middle-income status, primarily due to its limited R&D spending and dependence on government initiatives ( Park and Kim, 2021).

The Lagos Plan of Action, established in 1980, laid the groundwork for the subsequent Lagos Consolidated Plan of Action, which emerged in the early 2000s. This latter plan underscored the significance of Science, Technology, and Innovation (STI) as essential components for fostering socio-economic advancement across African nations ( Khan, 2022; Yongabo & Göransson, 2020). Key objectives of these initiatives included enhancing research capabilities and reforming the higher education landscape. A notable commitment was made for African countries to allocate 1% of their Gross Domestic Product (GDP) towards research and development ( Khan, 2022). The evolution of innovation and science and technology policies in developing nations is being influenced by the rise of a knowledge-based economy, the forces of globalization, and the involvement of various international organizations, as illustrated by the experiences of the BRIC countries ( Gachie and Govender, 2017). However, STI institutionalization in Africa has not kept pace with other regions; many African countries lack sufficient STI management bodies or policies to advance STI successfully. Developing countries, especially those in sub-Saharan Africa, often regard their National Innovation Systems (NIS) as “immature” or “catching up” ( Skorge, 2024; Yongabo & Göransson, 2020; Seifu et al., 2022).

In the African context, Science, Technology, and Innovation Policies (STIP) can be reconceptualized as a mechanism for transforming the continent’s rich history, cultural heritage, and artisanal practices into globally recognized creative industries. This would change the way STI is conceptualized and create an ecosystem similar to Silicon Valley, which creates millions of jobs while balancing “grassroots” and “mission-oriented” innovation policies to boost national innovation capacities, encourage inclusive growth, and combat poverty and inequality ( Khan, 2022; Mavhunga, 2017). Evidence showcases that while R&D significantly drives innovation, which in turn enhances firm productivity, the engagement in R&D and innovation is largely influenced by access to skilled labour and financial resources, highlighting the importance of developing mechanisms and policies to promote knowledge production and bolster innovation initiatives ( Keraga & Araya, 2023).

With South Africa and Egypt contributing the vast majority of contributions, Africa has the weakest research infrastructure of any continent. It also contributes less than 2% of global research publications, struggles with brain drain, and has a very low innovative profile, with 88% of patent activity concentrated in South Africa. Furthermore, Africa falls short of meeting the GERD target of allocating 1% of GDP for research and development ( Khan, 2022; Mavhunga, 2017). In this regard, the latest updates for Ethiopia, Kenya, and Rwanda showed Gross Expenditure on Research and Development (GERD) as follows Kenya (0.8%), Rwanda (0.7%), and Ethiopia (0.3%) ( Ogada et al., 2022). In Africa, significant development entities, including universities, research institutions, financial organizations, educational bodies, and intermediary organizations, are present; however, they often exhibit limited competencies. Furthermore, these entities frequently lack the necessary connections and institutional capacity to effectively participate in innovation strategies that are responsive to market demands ( Seifu et al., 2022).

## Methods

The study employed a qualitative approach, specifically document review as the primary methodology to conduct a thorough evaluation of the existing Science, Technology, and Innovation (STI) policies and legal frameworks in Ethiopia within the context of the Regional Innovation System (RIS). The study’s primary objective is to identify possibilities and gaps that could guide future policy while analysing how well STI laws and policies connect with the National Innovation System (NIS). The data collection process consisted of a review of relevant documents, which were categorized into four key areas: government publications, including significant policy documents such as the National Science and Technology Policy (2012) and the Investment Proclamation (2020); reports from international organizations like the United Nations and the World Bank; academic literature that examines STI policies in Ethiopia alongside comparative studies in emerging economies; and regional and global initiatives, including frameworks like the Sustainable Development Goals (SDGs).

In terms of the inclusion criteria, the primary one is how relevant policies and legal frameworks are to the national innovation system. Science, Technology, Innovation (STI) policies and legal frameworks, and their impact on Ethiopia’s economy and society are the subjects of the selected documents. The process of reviewing the documents was stopped when it became clear that adding more documents to the study was no longer adding anything either novel or relevant. Each document is legally sourced from official government ministries. Although the exclusion criteria weren’t mentioned explicitly, it appears from the selection process that documents that did not match the criteria for inclusion would be excluded.

The selected documents cover a wide range of important topics that are crucial to fostering creativity, technological advancement, and economic growth in Ethiopia. The National Science and Technology Policy (2012), the STEM Education Program (2016; Investment Proclamation (1180/2020), the Intellectual Property Law (320/2003; National Research and Development Strategy (2021), the Industrial Parks Development Proclamation (886/2015), the Digital Strategy (2021), the Addis Ababa University Innovation and Incubation Policy (2022), the Professional and Program Mix Policy (MoE, 2008), the Higher Education Proclamation (650/2009), and Directive No: Research (01/2019) are among the current STI policies and legal frameworks covered in the study.

Data analysis involved a deductive approach to thematic analysis was used in the study’s methodology, with a focus on predetermined categories such as STI policies and legal frameworks, innovation opportunities, policy gaps and challenges, and comparative analysis of Ethiopia’s National innovation ecosystem capabilities with Kenya and Rwanda. This thematic analysis was conducted enabling a comprehensive exploration of the relationships between STI policies, National and regional innovation dynamics, and economic outcomes. Triangulation was used to compare data from multiple sources, such as government publications and scholarly literature, in order to increase the results’ credibility. Furthermore, input from subject-matter specialists was requested to confirm interpretations drawn from the examined materials. Notwithstanding the comprehensiveness of this document assessment, some limitations were noted, including possible gaps brought on by the use of publicly accessible materials and the constantly evolving nature of policies. The study’s findings are intended to enhance knowledge of how STI policies impact Ethiopia’s national innovation system and support sustainable development.

### Ethiopia, Kenya, and Rwanda Experience

The Global Innovation Index (GII) serves as an annual evaluation that measures the innovation capabilities and achievements of economies across the globe. It includes more than 80 metrics categorized into seven distinct groups: institutions, human capital and research, infrastructure, market sophistication, business sophistication, knowledge and technology outputs, and creative outputs. According to the 2023 Global Innovation Index, each country exhibits distinct characteristics.

Ethiopia, a low-income country, ranks 125th with a population of 123.4 million, a GDP of 347.8 billion PPP$, and a GDP per capita of 3,434 PPP$. Kenya, on the other hand, is classified as a lower-middle-income nation, has 54 million people, a GDP of 311.8 billion PPP$, a GDP per capita of 6,122 PPP$, and a rank of 100. Similarly, Rwanda, which is placed 103rd and with a population of 13.9 million, a GDP of 37.6 billion PPP$, and a GDP per capita of 2,836 PPP$, is likewise categorized as a lower-income country. Based on the Global Innovation Index, this report presents a comparative picture of these three countries.

Ethiopia, Kenya, and Rwanda exhibit significant differences in their institutional frameworks, which have an effect on their national innovation systems. Ethiopia has a very low total institutional score of 32.7, particularly when it comes to the business environment (30.5) and institutional environment (18.6), which restricts Ethiopia’s capacity to foster innovation. Kenya, on the other hand, had a score of 45.0, which suggests that it has made some progress in terms of innovation but still needs to improve. This can be attributed to a somewhat supportive corporate climate (30.5) and a more favorable regulatory environment (49.0). With a remarkable overall score of 65.4, Rwanda leads the country in innovation. This is driven by a robust business climate (79.1) and a sound regulatory framework (63.2). Considering these disparities, it is advisable that Ethiopia and Kenya take lessons from Rwanda’s effective policies in order strengthen their institutional frameworks and enhance their business environments, so enhancing their ability to foster innovation. Promoting local innovation systems and promoting economic growth requires policymakers to have a solid understanding of the institutional context. Globally, Singapore, Switzerland, and Finland had the highest marks in the world for the institutional structure (98.4, 87.3, and 85.4, respectively).

Rwanda scored 22.6, higher than both countries, indicating a strong emphasis on R&D (3.5) and significant expenditures in education (37.7) and higher education (26.6). Ethiopia may need to improve its research and tertiary education programs in order to increase its capacity for innovation and national competitiveness. This analysis emphasizes how important research and education are to fostering creativity. Both Rwanda and Kenya are improving their R&D and human capital frameworks. The differences in these countries’ R&D and educational strategies highlight the necessity for purposeful advancements in human capital development to promote national progress by demonstrating how varying levels of investment and emphasis may affect their whole innovation ecosystems. Sweden, Singapore, and South Korea have the highest human capital scores in the world, at 66.9, 63.2, and 62.7, respectively.

There are significant disparities between the infrastructure landscapes of Ethiopia, Kenya, and Rwanda that have implications for their distinct national innovation systems. There is potential for improvement, as evidenced by Ethiopia’s 12.1% overall infrastructure score, particularly in the Information and Communication Technologies (ICT) category, where the country scored 17.4. In contrast, Kenya has a stronger infrastructure base, scoring a respectable 25.3 and an impressive 56.4 in ICTs, demonstrating the nation’s advanced digital ecosystem and the government’s commitment to online services and e-participation. Rwanda leads the field in this comparative analysis with an infrastructure score of 27.9, which highlights its efficient use of ICTs at 53.7, and a general infrastructure score of 18.3, which demonstrates its strategic investments in technology and development. Ethiopia may find it challenging to promote innovation due to its relatively underdeveloped infrastructure, whereas Kenya and Rwanda, with their more robust infrastructure, are better positioned to lead in national innovation, enabling greater connectivity, sustainable ecological practices, and increased entrepreneurial activity. With scores of 69.2, 67.6, and 65.6, respectively, Finland, Sweden, and Denmark are the top three nations in the world for infrastructure.

A comparison of Rwanda, Kenya, and Ethiopia’s market sophistication reveals several intriguing distinctions that have significant implications for each country’s innovation processes. Ethiopia has a 19.8 market sophistication score, compared to Rwanda’s 18.6 and Kenya’s higher 22.1 scores. To be more precise, Kenya scores 21.5, which indicates that it has a strong investment performance and offers a conducive atmosphere for funding innovation. Ethiopia’s investment score, however, lower at 0.4, emphasizes difficulties in bringing in both foreign and domestic funding, which may impede the development of creative businesses. Kenya is probably positioned to maximize its potential for regional competitiveness, although differences in investment and general market sophistication could result in diverse innovation paths. On a global scale, the leading countries in market sophistication are the USA, Hong Kong, and the United Kingdom, with scores of 82.9, 71.8, and 69.3, respectively.

Ethiopia’s business sophistication score of 14.7 indicates difficulties in creating a strong innovation environment. Nonetheless, with a score of 24.2, Kenya tops the list, demonstrating a higher ability to integrate knowledge workers and create innovative connections—two factors that are essential for promoting innovation and technological growth. Rwanda’s business sophistication score of 20.0 indicates a well-rounded strategy, bolstered by its focus on knowledge absorption (26.7) and innovation connections (24.9). Kenya appears to have a more dynamic environment for promoting innovation, which can propel economic development more successfully than Ethiopia’s existing situation, based on its better ratings for knowledge workers (22.7) and innovation links (23.2). On a global scale, Sweden, the USA, and Singapore lead in business sophistication, with impressive scores of 78.5, 69.9, and 65.6, respectively.

The comparative analysis of the knowledge and technology outputs among Ethiopia, Kenya, and Rwanda reveals distinct. Ethiopia’s knowledge and technology outputs stand at 17.9, with a particularly strong emphasis on knowledge creation (19.2) and knowledge impact (24.1). In contrast, Kenya demonstrates slightly higher overall outputs at 18.4, with significantly higher levels of knowledge diffusion (20.4) compared to Ethiopia and Rwanda.

Rwanda’s strategy appears to be centered on optimizing the impact of current information, whilst Kenya and Ethiopia are concentrating on using knowledge creation and impact to spur innovation. A more competitive and connected national economy might result from improving the innovation ecosystems of all three countries through regional cooperation and focused investments in knowledge creation and diffusion, as indicated by the disparate performance in these indicators. Switzerland, the United States, and Sweden are the top three countries in terms of knowledge and technology outputs, scoring 65.3, 63.9, and 63.4, respectively.

Ethiopia, Kenya, and Rwanda exhibit noteworthy disparities in their creative outputs and innovation skills, which have important ramifications for their respective national innovation systems. Ethiopia’s creative output score, as the data shows, is 4.5, with intangible assets accounting for the majority of this score (2.1) and online creativity being a relatively new sector (13.6). Kenya, on the other hand, produces significantly more creative work (14.1), supported by significant intangible assets (18.9) and a robust online creativity score (17.2). Rwanda’s dedication to promoting a digital economy is demonstrated by its strengths in intangible assets (7.0) and online creativity (12.2), while having a lower overall creative output (6.9). Ethiopia and Rwanda must improve their intangible assets and digital creativity programs to promote innovation, as these discrepancies underscore Kenya’s highly developed creative economy. The implications for the national innovation system are significant; improved cooperation between these countries may make it easier to exchange information and distribute resources, which would eventually lead to a more resilient and cohesive creative economy throughout East Africa. Switzerland, the United Kingdom, and Hong Kong lead the global GII in terms of creative outputs and innovative capabilities, with scores of 68.5, 60.0, and 59.2, respectively.

Ethiopia, Kenya, and Rwanda have established legal institutions to coordinate STI activities, with the Ministry of Science and Technology in Ethiopia, the National Council of Science and Technology in Kenya and Rwanda, and the Kenya National Innovation Agency in Kenya. The human resource capacity varies across countries, with Ethiopia and Kenya having a majority of personnel in agriculture and veterinary sciences, while Rwanda has a majority in natural sciences. The majority of personnel are from government institutions in Ethiopia (70.1%) and Rwanda (56.1%), while in Kenya, they are from higher education institutions (56.7%). Rwanda has made progress in STEM education, with 22.4% student enrollment, but female students make up less than a third of this total. The countries have made progress in establishing centres of excellence under the Africa Centre of Excellence program and industrial parks, with Ethiopia leading the region with five industrial parks. There is a pressing necessity to strengthen collaborations between countries, enhance infrastructure, encourage investments from the private sector, and cultivate human resources, particularly emphasizing STEM fields and the participation of women, in order to improve the execution of science, technology, and innovation (STI) initiatives.

### Policy and legal frameworks in Ethiopia

The National Science and Technology Policy, established in 2012, serves as a foundational framework aimed at promoting research, development, and innovation within the nation. It promotes a conducive ecosystem that will support innovation, research, and technology as engines of economic growth and sustainable development. It emphasises public-private partnerships in technology development and commercialisation, strengthens the capacity and efficiency of the research and innovation ecosystem, and creates an environment that is conducive to technology transfer and intellectual property protection. According to the STI Policy, research is an essential part of a strategic plan to effectively adopt foreign technologies, modify them for the local context, employ them successfully, and share knowledge through connections between academia and industry ( Mamo, 2015).

The outcomes of research projects are expected to influence the advancement of the country.

A STEM education program was launched by the Ethiopian Ministry of Education in 2016 in an effort to enhance the country’s educational system. STEM seeks to improve the quality of education in these fields and give students the skills they need to be successful in the contemporary workforce. The program plays an important role in promoting innovation and furthering the nation’s technological advancement. Through STEM education, the initiative hopes to develop a workforce that can promote innovation and further the expansion of a knowledge-based economy.

The Investment Proclamation (1180/2020), which was issued by the Ethiopian Investment Commission (EIC), provides investors with protection, assurances, and incentives that motivate them to engage in different areas of the Ethiopian economy. The commission acts as a point of contact between the business community and government organizations, providing direction, encouragement, and assistance throughout the investment process. The Investment Proclamation serves as a tool for the national innovation system, encouraging creativity and technology transfer by providing incentives for investments in vital sectors like as R&D, innovation, and technology. The declaration also encourages the use of innovative technologies and the development of innovative businesses.

The Intellectual Property Law (320/2003) of Ethiopia provides researchers, inventors, and entrepreneurs with the legal tools to protect their intellectual property in order to foster technological advancement and innovation. The proclamation outlines the procedures for registering and defending intellectual property rights in Ethiopia and ensures that business owners and innovators have the exclusive right to their endeavors, including designs, ideas, and works. Enforcing intellectual property rights ensure that creators may make money off of their creations and encourages more investment and creativity in the country, all of which help to create an atmosphere that is favorable to innovation.

A National Research and Development Strategy was passed by Ethiopia in 2021 with the goal of promoting technical advancement, innovation, and research across a number of domains. The strategy outlines the major R&D priorities, which include raising educational standards, promoting industry-academia collaborations, and building out the research infrastructure. The ministry intends to employ research and innovation to address societal concerns, encourage sustainable economic growth, and increase national competitiveness by focusing on three areas.

The National Research and Development Strategy offers a road map for orchestrating R&D activities with national priorities, encouraging stakeholder partnerships, and optimising resources to foster economic growth and innovation. Through the explicit articulation of particular goals and practical strategies, the strategy facilitates the creation of a favourable environment that supports research and development activities, stimulates the creation and sharing of information, and helps to commercialize research findings. Employing these initiatives, the ministry strives to strengthen Ethiopia’s innovation ecosystem, cultivate a research and innovation culture, and propel sustainable growth within the nation.

Ethiopia enacted the Industrial Parks Development Proclamation (886/2015) to inspire the foundation of industrial parks throughout the nation to foster industrialisation and economic growth. The proclamation lays forth guidelines for designing, building, and operating industrial parks to bring in foreign and domestic capital, encouraging local businesses to integrate into global value chains and create employment opportunities. Beyond and above, the proclamation aspires to promote manufacturing operations, boost exports, and advance Ethiopia’s socio-economic development by offering incentives and infrastructure support to companies working within industrial parks.

Creating a digital strategy for Ethiopia (2021) is crucial to leveraging the capacity of technology to boost the national economy, enhance social services, and generate employment.

The National Innovation System must be taken into consideration while formulating a digital strategy for Ethiopia, as must the establishment of a collaborative ecosystem that can capitalize on the advantages enjoyed by the government, business, academia, and civil society. Ethiopia has the capacity to create a vibrant innovation ecosystem that fosters cooperation and information sharing, accelerating digital transformation and economic advancement. This plan comprises investing in R&D, supporting tech companies, and fostering a creative culture that values creativity and entrepreneurship. Through the alignment of digital initiatives with national priorities and the utilization of current resources, Ethiopia can establish itself as a frontrunner in the digital economy and promote sustainable and inclusive growth for all of its residents.

An innovation and incubation policy was formed by Addis Ababa University in 2022 with the goal of supporting the growth and prosperity of start-ups and small enterprises in the technology industry. The strategy states that the incubators provide new companies with access to resources such as office space, capital, networking opportunities, and mentorship in a supportive setting. Conversely, innovation policies offer frameworks and tenets to promote technology transfer, research and development, and entrepreneurship. Strong innovation policies combined with technological incubation can help nations create an environment that is favorable to innovation, economic growth, and increased global competitiveness. Policies for innovation and technology incubation are essential for fostering technology transfer, university-industry connections, and the growth of local talent.

The Professional and Program Mix Policy (MoE, 2008) provides a comprehensive examination of the annual intake, enrollment growth, and professional program mix in Ethiopian public higher education institutions. It provides a detailed explanation of the 70:30 undergraduate-professional balances that prioritizes science and technology over the arts and social sciences. This strategy has implications for industry-university collaboration on research and training. This policy’s potential impact on Ethiopia’s national innovation system makes it relevant.

The Higher Education Proclamation (650/2009) serves as the fundamental legislative framework governing the education system at the national level. This legislation outlines the establishment, functioning, and regulatory compliance of higher education institutions within the country. It sets forth standards for curriculum development, accreditation processes, academic autonomy, and the administration of higher education. The primary goals of the Higher Education Proclamation include ensuring the delivery of high-quality education, fostering innovation and research, and cultivating a skilled workforce capable of contributing to the socioeconomic development of the nation. The proclamation mandates universities to identify their main research areas and themes during conversations with key stakeholders, taking into account the nation’s top priorities and the institution’s competitive advantages; university partnership on research schemes with other domestic and foreign institutes, research centres, and businesses is authorised under the proclamation; the proclamation makes it apparent that collaborations with business are important for research and technology transfer.

The implementation of directives concerning research, technology transfer, university-industry collaboration, and community services for higher education institutions in Ethiopia, as outlined in Directive No: Research 01/2019, aims to strengthen the research and innovation capacities of these institutions. It is clearly stated under the preamble as:

“ *It is mandatory to put in a place and coordinate a well-developed system or research, technology transfer, university-industry linkage, and community services to contribute in ensuring sustainable and holistic development across the nation.”* p1

The objective of the directive is to cultivate an environment of innovation and cooperation among higher education institutions, which will ultimately result in the creation of new technologies, products, and services that serve the broader interests of society. Through fostering cooperation between researchers and business partners and fostering community involvement, these programs seek to boost economic growth and strengthen the area’s competitive advantage. Most significantly, the order pushes academic institutions to use grants and seed funds to commercialize discoveries developed in their labs and departments.

### SWOT-TOWS Analysis of Policies and Legal Frameworks (RRI-AIRR framework)

The notion of Responsible Research and Innovation (RRI) has been a prominent scientific policy framework in the past ten years (Samara et al., 2024). Public engagement, science education, ethics, gender, open access, and governance are the six structuring themes of RRI, which is regarded as a policy framework in which various national innovation actors, including businesses, civil society organizations, researchers, individual citizens, and policymakers, team up in order to better align processes and outcomes with the needs, values, and expectations of society (Andronikidis et al., 2025; Fitjar et al., 2019). It has been presented as a framework to promote both ideas at the regional level and to connect research and innovation with broader social ideals (Bidstrup et al., 2024). Informed, inclusive, sustainable, and responsible regional development is the goal of this partnership (Burget et al., 2017). In this context, the Anticipation, Inclusiveness, Reflexivity and Responsiveness (AIRR) dimensions of governance, have gained particular importance as a base for enhancement of the RRI framework at territorial level (Andronikidis et al., 2025; Stilgoe et al., 2020). Thus, adopting RRI themes and AIRR dimensions, the study examined the current Science, Technology, and Innovation (STI) policies and regulatory frameworks. Lastly, the following table offers the Strength, Weakness, Opportunity, and Threats analysis (SWOT-TOWS).

**Table 1.** SWOT analysis.

	Strength	Weakness
**Internal**	• Promoting the ecosystem for research and innovation: R&D, innovation, and technology transfer are specifically highlighted in the National Science and Technology Policy, National Research and Development Strategy, and directives (such as Directive No: Research 01/2019). • Legal Foundation for IP Protection: The Intellectual Property Law (320/2003) offers a foundation for intellectual property protection, which is essential for supporting innovation and drawing in capital. • Development of Public-Private Partnerships: To support technological research and commercialization, a number of policies (such as the National Science and technological Policy) promote collaborations between government, business, and academia. • Investment Incentives: To promote investment, particularly in the technology and research and development industries, the Investment Proclamation (1180/2020) provides incentives that may spur innovation. • Targeted Approaches: Certain programs, such as STEM Education (2016), fill skills shortages and advance a knowledge-based economy. • Infrastructure development: Manufacturing operations are supported and industrialization is encouraged by the Industrial Parks Development Proclamation (886/2015).	• Bureaucratic Setting: Despite investment incentives, bureaucratic and regulatory obstacles may still exist and impede the rate of innovation. • Implementation Gaps: Although policies may exist in theory, they may not be properly applied or enforced. • Limited R&D Funding: Adequate funding for infrastructure, innovation, and research is essential to the success of these initiatives. • Coordination Issues: There is need for improvement in the way various government departments, academic institutions, and the commercial sector coordinate with one another. • Skills Gap: Although there are STEM programs in place, closing the skills gap may take time and money. • Misalignment of Industry-Academia: It may be difficult to close the gap between scholarly research and practical industrial use. • Technology Access: Obstacles may arise from issues with the availability and cost of technological infrastructure and resources.
**External**	**Opportunities**	**Threats**
	• Enabling Global Partnerships: The policies make it possible to work with companies, funders, and research institutions around the world. • Promoting Entrepreneurship: By offering resources like money and incubation facilities, policies can encourage an entrepreneurial culture, particularly in tech businesses. • Attracting Foreign Investment: Opportunities to draw in foreign investment in technology and R&D are made possible by robust IP regulations and investment incentives. • Digital Economy: Ethiopia is able to take use of the digital economy's potential for economic transformation thanks to its digital strategy. • Sustainable Development: Research that is in line with national priorities creates opportunities for innovative solutions to societal problems. • Promoting Industry-Academia Collaboration: Technology transfer and commercialization can be facilitated by robust policy support for industry-academia cooperation.	• Political Instability: Investor confidence can be damaged and long-term strategy implementation impeded by political instability or inconsistent policies. • Financial Issues: Funding for R&D and innovation projects may be restricted by economic downturns or volatility. • Fierce global competition must contend with competition from both African and international countries to draw in investment and promote innovation. • Global Economic Disruptions: Changes in the world economy may have an impact on foreign investment and finance. • A shortage of qualified staff: The impact of the programs may be lessened by a lack of qualified workers, especially in the STEM fields. • Rapid technological change: Because of the speed at which technology is developing, policies and initiatives may need to be adjusted on a regular basis.

**Table 2.** TOWS matrix.

Strengths - Opportunity Strategies	Strengths - Threats Strategies
• Promotion of investment and use of the intellectual property incentive to draw foreign direct investment in technology and innovation where the country has its competitive advantage, such as in energy, tourism, and agriculture. • Building capability and enhancing innovation by means of international innovation initiatives. • To foster technology and knowledge transfer and commercialize research findings, national innovation actors and their network should be strengthened. • Promote tech start-ups and entrepreneurship by utilizing the digital strategy. • Strengthening the digital platform and strategy to support tech start-ups and entrepreneurship.	• To attract international investment, make use of government incentives and intellectual property laws. • Create connections and provide incentives to take advantage of chances to collaborate with the international community. • Boost tech start-ups and entrepreneurship by leveraging the digital strategy.
**Weaknesses - Opportunity Strategies**	**Weakness - Threat Strategies**
• Fostering the innovation system by bringing the players together to ensure that laws and policies are applied correctly. • Strengthening the financial system to fill a deficit in R&D and innovation efforts, with a focus on projects that support national development objectives. • Enhancing workforce skill and graduates' employability through dedicated educational initiatives. • To encourage investment and innovation, simplify laws and lower administrative barriers. • Develop and commercialize research discoveries, academic and business ties should be strengthened.	• Close the skills gap by implementing education and training initiatives that concentrate on fields with strong market demand. • To promote investment and innovation, simplify regulations and reduce administrative roadblocks. • Provide incentives and cultivate connections to open doors to collaboration with the international community.

### Opportunities for National Innovation System (NIS)

The Ethiopian National Innovation System (NIS) has the unrealized potential to use innovation, science, and technology to greatly improve economic growth, social development, and global competitiveness. By leveraging its strengths, addressing its present issues, and promoting collaboration in and outside the nation, Ethiopia may become a hub for innovative excellence and advance the African continent’s innovation scene. Ethiopia has the potential to play a significant role in determining the direction of science, technology, and innovation in the area through collaborations, strategic investments, and the implementation of laws that encourage innovation and entrepreneurship. Ethiopia has made significant strides in creating an environment that is supportive of innovation through the use of laws, programs, and alliances that facilitate knowledge sharing, technology transfer, and research. Science and technology have developed a strong basis as a result of the establishment of research institutes, incubation centers, and innovation hubs that serve as catalysts for entrepreneurship and innovation.

Ethiopia’s national development is influenced by a number of important elements. The nation’s strategic location in the Horn of Africa and plenty of natural resources are its main advantages, as they allow it to be close to the Middle East and its rich markets. Ethiopia’s ability to develop a robust economy and reach lower-middle-income status by 2025 is highlighted by the country’s impressive economic growth over the last 20 years. The creation of a National Innovation System (NIS) has been actively sought by the government, which has done so by creating a number of laws and policies pertaining to STI. The adoption of successful STI policies meant to broaden and expand the academic-industry relationship, the expansion of funds for research and development, the improvement of institutional capacity, and the economy.

Ethiopia has made structural restructuring, industrialization, and urbanization its top priorities in line with its goal of becoming a middle-income country by 2025. This emphasis has made it possible for the nation’s energy, transportation, and telecommunications infrastructure to advance significantly. This economic strategy’s main component is a large public investment in infrastructure, which attempts to close current gaps and foster an atmosphere that will support future private sector growth. Most notably, the Ethiopian government has been aggressively constructing industrial parks to boost the nation’s overall economic growth by exporting manufactured goods that generate foreign exchange, transferring technology, knowledge, and skills, and creating productive jobs for the people ( Jote, 2020).

Initiatives are also underway to establish linkages between industrial parks and universities across the nation. Most industrial parks have been located adjacent to universities to accommodate large-scale industrialisation projects. The Hawassa Industrial Park, for example, was built next to Hawassa University and is home to important domestic and international clothing and textile companies with 60,000 workers and an export revenue objective of over US$1 billion. The industrial parks of Bole Lemi-I and II, along with the Kilinto Industrial Park situated in the suburbs of Addis Ababa, demonstrate the broader network of industrial zones across Ethiopia, which includes locations in Dire Dawa, Mekelle, Adama, Kombocha, Jimma, Bahir Dar, Debre Birhan, and Aysha Dewalle ( Salmi et al., 2017). In summary, these industrial parks have played a crucial role in advancing Ethiopia’s industrial landscape by generating employment opportunities, enhancing government revenue and export levels, diversifying the range of industrial products, and attracting both Foreign Direct Investment and foreign exchange ( Jote, 2020).

### Challenges in Promoting National Innovation System (NIS)

Meaningful STI policymaking cannot be developed, carried out, monitored, or modified without an in-depth understanding of the nation’s context, strengths and weaknesses of the nation’s Science, Technology, and Innovation (STI) agents, their interactions, and incentives as well as barriers that they encounter, all of which are extremely country-specific ( Adenle et al., 2023). The obstacles of scaling up Ethiopia’s innovation ecosystem to keep up with the demands of an increasingly shifting global market persist despite advancements in science, technology, and innovation ( Liche & Střelcová, 2023; Gashahun, 2020). The inability to successfully implement the policy is one of the biggest obstacles. The majority of top-down policies have problems with execution. The lack of programs that contributed STI policy objectives into practice and the “Top-Down” approach to policy formulation have hindered efforts to implement the policy effectively, according to Agu et al. (2021). The lack of programs also adds to the inefficiencies in the implementation processes.

Effective policymaking in the field of science, technology, and innovation (STI) requires a thorough grasp of a country’s unique context, which includes the advantages and disadvantages of its STI stakeholders, as well as their interactions, incentives, and obstacles, all of which are influenced by the particular circumstances of that country ( Adenle et al., 2023). Ethiopia has made great strides in science, technology, and innovation, but there are still major challenges in developing its innovation ecosystem to meet the needs of a rapidly evolving global market. Ineffective policy translation into practical practice is a major problem that is frequently caused by a top-down strategy and a lack of public awareness. This problem is further worse by the lack of concrete action projects ( Agu & Ndinda, 2021).

Moreover, there is a significant lack of coordination and cooperation between the several National Innovation players and the scientific, technology, and innovation ecosystem’s stakeholders. Despite the effective partnerships between government agencies, research institutions, academia, and industry are essential to fostering innovation, knowledge exchange, and technology transfer, the Ethiopian Innovation ecosystem is not conducive and well-orchestrated ( Tekle, 2024; Kebebe, 2019). The STI policy aims to facilitate linkages among stakeholders, but in practice, there is immature coordination and collaboration, resulting in a disconnect rather than synergy, despite its commitment ( Agu & Ndinda, 2021).

Robust advancement in research and development is thought to promote science, technology, and innovation while also boosting competitiveness and economic growth. Research leadership and administration (motivation, teamwork, salary, external influence, political burden, lack of clear vision), communication and outsourcing of research (lack of funds, lack of communication, lack of industrial linkage, lack of partnership, lack of trained journalists), and lack of national strategy & policy (lack of information centre, lack of funds, lack of teamwork, lack of national research centre) are some of the concerns regarding research outputs ( Tesfa, 2015). Furthermore, Gelata et al. (2022) examined e-commerce in Ethiopia and noted that the country’s business had considerable hurdles, including a lack of infrastructure, a lack of trust, security risks, and a lack of a regulatory framework. The Ethiopian industry’s barriers include information technology, inadequate personnel training and expertise, a deficiency in IT infrastructure, customer awareness, and behaviour. The absence of well-structured data warehouses, coupled with inadequately designed websites that provide minimal or no information regarding stakeholders, significantly hinders the understanding of advancements within the sector ( Agu & Ndinda, 2021). Outdated laboratory facilities, insufficient equipment, and above all the poor telecommunication networks in Ethiopia contribute to the poor performance.

The lack of financial resources is the most significant barrier to STI development in developing countries, and Ethiopia is no exception. The critical challenges associated with funding have been categorized into two primary issues. First, there is a significant dependence on state funding coupled with inadequate involvement from the private sector, which has severely hindered science, technology, and innovation (STI) initiatives ( Gobena et al., 2021). Second, the lack of various nationally established guidelines and research evaluation frameworks for the distribution of limited financial resources constitutes an additional obstacle, contributing to inefficiencies and the misallocation of resources ( Agu & Ndinda, 2021).

Concerning the University-industry linkage (Partnership), several efforts exist to create a conducive learning environment for actors to engage in partnerships. Partnerships among National Innovation actors, the status of collective learning among actors is very low in Ethiopia is very low, particularly the roles of R&D institute, TVET College, and University Industries Linkage are very low in collaboration ( Gobena et al., 2021; Ayenew & Teklay, 2017; Ayenew, 2015). In spite of the government’s efforts to enhance and maintain a productive linkage between university and industry, the relation remains in its early developmental stage, with a significant number of outstanding tasks yet to be addressed ( Degaga & Senapathy, 2021).

### Policy Implication

The role that Science, Technology, and Innovation (STI) policies and legal frameworks play in strengthening Regional Innovation Systems (RIS) and enhancing regional competitiveness has become realised progressively in recent years. Science, Technology, and Innovation (STI) policies significantly determine global, regional, and national social and economic development.

Science, Technology, and Innovation (STI) policies significantly determine global, regional, and national social and economic development. The increasing reliance on knowledge-based innovation was a major factor in the formal recognition of this role in the later half of the 20th century. The relationships between various ecosystem players, including governmental organizations, educational institutions, research and development organizations, and industry sectors, have become crucial elements throughout this time ( Galvao et al., 2019).

Most significantly, building a robust and flexible STI ecosystem is a continuous process that necessitates constant collaboration, adjustment, and tuning. Thus, policymakers need to be flexible, willing to learn from ecosystem participants, and ready to alter their plans when new information becomes available or the situation calls for it. A thriving innovation ecosystem where businesses, scholars, and investors can collaborate effectively to produce new ideas and technologies depends on the creation of agile policies ( Ratanawaraha et al., 2024).

Science, Technology, and Innovation (STI) are important, and the Ethiopian government recognizes this. Therefore, in order to create possibilities to promote economic growth, it has implemented a number of STI policies over the years and developed organizations, plans, and initiatives in the main priority areas.

Ethiopia spends about 0.51% of its gross domestic product (GDP) on STIs, which is below the African Union’s (AU) recommended target of 1%. Several stakeholders participate in the STI ecosystem in Ethiopia, with the nature of participation ranging from policy development, regulation, and implementation of STI programs, initiatives, plans, and projects. The different stakeholders have different mandates and exert different levels of power and influence across the STI landscape.

Emerging economies depend on a combination of technological advancement and homegrown innovation to enhance their national innovation systems and policy frameworks, thereby maximizing the advantages of global knowledge transfer for science, technology, and innovation collaboration. This is because many of these nations want to achieve economic growth and competitiveness. Industry, academic institutions, and research centres must change to build mutually beneficial partnerships and complement each other’s strengths to achieve the goals of STI cooperation. Collaborations ought to be established in critical domains such as institutional policy and governance frameworks, higher education in science and technology, entrepreneurship, research infrastructure, legal systems, and technology transfer ( Adenle et al., 2023). The adoption of incremental innovations, prioritizing innovation in sectors with significant economic impacts, concentrating on demand-oriented innovation, implementing spatial innovation, and creating a governmental coordination platform for development priorities and collaboration among ecosystem factors are the five main directions proposed by Shkabatur et al. (2022) for innovation and entrepreneurship policy. These strategies aim to enhance economic growth, improve productivity, and generate jobs.

The key to maximising the effectiveness of the national innovation system is to strengthen research by creating substantial and long-term funding sources, safeguarding the research council already in place, endorsing the professional association, and figuring out how to leverage the tremendous amount of knowledge and expertise held by professionals from Ethiopia’s diaspora ( Tesfa, 2015). To promote economic growth and optimise Ethiopia’s economy’s competitiveness, the national innovation actors ought to be motivated to engage in R&D and learning initiatives, whether by creating new ones or modifying existing ones ( Gobena & Kant, 2022)

Policymakers in Ethiopia ought to focus on increasing the number of citable journals, encouraging patent registrations, and promoting technology exports within the context of research and development (R&D) innovation to stimulate economic growth ( Agezew, 2024). Merely increasing research funding may prove insufficient for economic advancement unless it is paired with targeted R&D initiatives that have a direct economic impact. This underscores the necessity for policies that facilitate collaboration among government entities, academic institutions, and the private sector to enhance innovation and technology transfer. A supportive policy framework, the expansion of industries, the enactment of intellectual property rights protection laws, a burgeoning economy, the establishment of science and technology universities, the development of industrial parks, and improvements in infrastructure represent significant opportunities ( Degaga & Senapathy, 2021). Reviving university-industry links (UIL) and addressing the aforementioned issues require a concerted effort from government agencies, academic institutions, businesses, and other pertinent players. Additionally, it is critical to take use of the newly discovered opportunities and to fortify the supportive environment so that university industry linkages can flourish with greater vigour.

Effectively optimizing the country’s Science, Technology, and Innovation (STI) policies is essential to promoting economic growth and raising competitiveness. Improving operational efficiency, reducing expenses, and increasing productivity can all help to achieve. STI policies have the potential to have a significant positive impact on the overall economy. Ethiopia must therefore work to maximize STI’s potential to boost its social well-being and economic prosperity, and this is crucial. The current state of affairs indicates a disparity in the effective implementation of the aforementioned efforts to establish a National Innovation System (NIS) and to develop comprehensive policies on Science, Technology, and Innovation (STI).

## Conclusion

Ethiopia’s pursuit of innovation, economic growth, and increased competitiveness depends heavily on the laws and regulations governing science, technology, and innovation (STI). Research, entrepreneurship, technology transfer, and collaborative learning are all made possible by these frameworks. The National Science and Technology Policy, the Strategic Policy for National Science, Technology, and Mathematics Education, the Investment Proclamation, the Intellectual Property Law, the National Research and Development Strategy, the Industrial Parks Development Proclamation, the Digital Strategy for Ethiopia, the Technology Incubation and Transfer Policy, the Professional and Program Mix Policy, and the Higher Education Proclamation are just a few of the policies that have been put in place to ensure the Ethiopian National Innovation System operates without interruptions. Ethiopia can strengthen its STI policies to support a National Innovation System (NIS) that fosters economic growth, technical advancement, and sustainable development by assessing current opportunities, resolving current difficulties, and proposing alternative policies.

Ethiopia has challenges in creating all-encompassing STI policies and successfully implementing them. The study investigated the ecosystem’s existing challenges. It emphasizes the importance of STI capacity-building for sustainable development and the necessity of utilizing innovative results within a strong policy framework. Policy gaps and the need for more robust institutional support are two issues that require attention. The laws and regulations relating to science, technology, and innovation (STI) are crucial for Ethiopia to pursue innovation, economic growth, and enhanced competitiveness.

These frameworks create an atmosphere that supports technology transfer, research, entrepreneurship, and collaborative education. A strong legal and policy framework is necessary for the Ethiopian National Innovation System to function effectively. This framework is established by the National Science and Technology Policy, the Strategic Policy for National Science, Technology, and Mathematics Education, the Investment Proclamation, the Intellectual Property Law, the National Research and Development Strategy, the Industrial Parks Development Proclamation, the Digital Strategy for Ethiopia, the Technology Incubation and Transfer Policy, the Professional and Program Mix Policy, and the Higher Education Proclamation that were established. Ethiopia can strengthen its STI policies to support a National Innovation System (NIS) that fosters economic growth, technical advancement, and sustainable development by assessing current opportunities, resolving current difficulties, and proposing alternative policies.

### Ethics and consent

Ethical approval and consent were not required.

## Data Availability

No data associated with this article. The article does not contain any associated data because it primarily focuses on the evaluation of existing Science, Technology, and Innovation (STI) policies and legal frameworks in Ethiopia rather than presenting original empirical research findings. It serves as a methodological discussion and analysis of the current state of STI in the context of the National Innovation system (NIS), emphasizing policy gaps and recommendations for improvement without generating new data. Therefore, no datasets are available for publication as the content centers on policy analysis and strategic insights rather than quantitative or qualitative research outputs. No extended data associated with this article.
