# Supplementary material for: National Innovation System (NIS) as a Means for Development: Policies, Opportunities, and Challenges in Ethiopia
Source: F1000Res. 2025 Feb 18;14:34. Originally published 2025 Jan 6. [Version 2] doi: 10.12688/f1000research.159772.2 (PMC12093090; doi:10.12688/f1000research.159772.2)
Supplement: Peer review history

# Reviewer report: Reviewer response for version 2

**DOI:** 10.5256/f1000research.178350.r381898

**Published:** 2025-05-20

**Authors:** Simon Ndicu

**Affiliations:** 1 Kenyatta University (Mama Ngina University College), Gatundu, Kenya

Thank you for the opportunity to read and review this thought provoking paper. A commend the authors for focusing on NIS especially from developing regions, where the concept of NIS has not been adequately investigated,

 By and large the paper has adequate scope, in my reviewers report, I will concentrate on the areas of improvement.

 The **introduction part** can be enriched further, after stating the objective of the papers, the authors, need to provide further justification on why the objectives are important why should we care about the study objectives. Secondly, the authors need to mention their unique contribution in this area of study. Lastly, towards the end of the introduction sections, it’s good to give us a glimpse of the paper organization, sections in the paper, methods, data and variables. So that by the time we are done reading introduction part we are able to grasp whole picture of the study. 

**Literature review**

 It seems the literature review has been divided into two parts *The* *National Innovation System (NIS)*, and *Science, Technology, and Innovation Policies (STIP) in Emerging Economies*

 My observation is that the literature on NIS is very brief mainly focusing on the importance of NIS. I advise the authors to look at the composition of NIS.  The current literature is at quintuple helix (recent works of EG Carayannis). I expect the authors discuss how the five helix are interconnected in Ethiopia.  Secondly, the authors need to mention on the measurement of NIS productivity by discussing the inputs and outputs of NIS (literature by Global innovation index GII). Lastly Factors hindering effective functioning of NIS, ought to be mentioned here in general.

***On the Science, Technology, and Innovation Policies (STIP) in Emerging Economies part***

 I expected the authors to discuss here regional and local  policies like ASTII ,  STISA 2014-24 and Agenda 2063, and describe how Ethiopia is catching and implementing this policies . I also expected to hear about Ethiopia STI policy implementation framework. Is there a commission STI, a national innovation agony or even a national research fund in Ethiopia?

**Methods**

 I suggest a table showing the four categories of the documents received, and the objectives would have been clearer. The readers may want know which documents were reviewed per study objective.

 Ethiopia, Kenya, and Rwanda Experience

 I did not see any justification provided for comparing innovation in these three countries given the fact that they don’t belong to the income level!! . I advise the authors to compare economic peers or all countries in eastern Africa region including others like Uganda Tanzania Sudan etc. the authors can also compare Ethiopia with other lower income countries in sub-Saharan Africa.

 Secondly, I advise the authors to use graphs to compare the countries, visual graphics,/maps are very effective in countries comparison.  

**Findings/in terms of opportunities and challenges**

 These finding I suggest they be presented in form of a table for clarity and focus – each opportunity or challenge be accompanied by the document reviewed.

Is the work clearly and accurately presented and does it cite the current literature?

Partly

If applicable, is the statistical analysis and its interpretation appropriate?

Partly

Are all the source data underlying the results available to ensure full reproducibility?

Partly

Is the study design appropriate and is the work technically sound?

Yes

Are the conclusions drawn adequately supported by the results?

Partly

Are sufficient details of methods and analysis provided to allow replication by others?

Partly

Reviewer Expertise:

National Innovation Systems, Economics of ST&I Programs, Innovation and Development

I confirm that I have read this submission and believe that I have an appropriate level of expertise to confirm that it is of an acceptable scientific standard, however I have significant reservations, as outlined above.

# Reviewer report: Reviewer response for version 2

**DOI:** 10.5256/f1000research.178350.r367300

**Published:** 2025-05-20

**Authors:** Elpida Samara

**Affiliations:** 1 University of Western Macedonia, Kozani, Greece

It is clear that the author has effectively addressed the major reviewer comments and substantially improved the clarity, conceptual focus, and methodological rigor of the paper.

 Key enhancements include the shift from a regional to a national innovation system framework (RIS to NIS), a clearer articulation of objectives, a better organized structure, and the addition of a SWOT-TOWS analysis to increase policy relevance.

 These changes demonstrate responsiveness to feedback and significantly strengthen the manuscript’s contribution to the field.

Is the work clearly and accurately presented and does it cite the current literature?

Yes

If applicable, is the statistical analysis and its interpretation appropriate?

Not applicable

Are all the source data underlying the results available to ensure full reproducibility?

Yes

Is the study design appropriate and is the work technically sound?

Yes

Are the conclusions drawn adequately supported by the results?

Partly

Are sufficient details of methods and analysis provided to allow replication by others?

Yes

Reviewer Expertise:

Innovation systems, regional development, entrepreneurship

I confirm that I have read this submission and believe that I have an appropriate level of expertise to confirm that it is of an acceptable scientific standard.

# Reviewer report: Reviewer response for version 2

**DOI:** 10.5256/f1000research.178350.r367299

**Published:** 2025-03-12

**Authors:** Shem Sikombe

**Affiliations:** 1 The Copperbelt University, Kitwe, Copperbelt Province, Zambia

Thank you so much for the opportunity to review the second version of the article.

 The authors have taken time to address the concerns which I raised in the initial review as follows:

 1. The objectives have been included in the revised version as suggested.

 2. The inclusion and exclusion criteria have been included in the methods section.

 3. The approach to thematic analysis is clear.

 4. The challenges in promoting RIS, opportunities and policy implications are well articulated in the revised version.

 Overall, the revised version has taken on the suggestions, and I have no further comments.

Is the work clearly and accurately presented and does it cite the current literature?

Yes

If applicable, is the statistical analysis and its interpretation appropriate?

Not applicable

Are all the source data underlying the results available to ensure full reproducibility?

No source data required

Is the study design appropriate and is the work technically sound?

Partly

Are the conclusions drawn adequately supported by the results?

Yes

Are sufficient details of methods and analysis provided to allow replication by others?

Partly

Reviewer Expertise:

Supply Chain Management, Research and Inovation, Knowledge Management, Operations Management and General Management.

I confirm that I have read this submission and believe that I have an appropriate level of expertise to confirm that it is of an acceptable scientific standard.

# Response

**Published:** 2025-03-12

**Authors:** Kassahun Sube

**Affiliations:** Development studies, Addis Ababa University, Addis Ababa, Addis Ababa, Ethiopia

Thank you very much!

# Reviewer report: Reviewer response for version 1

**DOI:** 10.5256/f1000research.175551.r360804

**Published:** 2025-02-11

**Authors:** Elpida Samara

**Affiliations:** 1 University of Western Macedonia, Kozani, Greece

The article requires substantial revisions to strengthen its overall structure, clarity, and coherence. These improvements are crucial for the manuscript to meet the indexing standards and increase its potential for successful acceptance. Specifically, the following areas need significant attention:  

 1. **Conceptual Clarification:**

 The focus of the paper should be clearly defined—whether it addresses regional innovation systems (RIS) or national innovation systems (NIS). The current overlap between these two perspectives creates conceptual ambiguity. Adding a section that explains the distinctions and linkages between the two systems is highly recommended.

 Alternatively, if the focus is on the national level, the title and literature should reflect that appropriately.

 To enhance the coherence and academic rigor of the study, I recommend the following adjustments:

 1. Add a dedicated section that defines and differentiates the concepts of Regional Innovation Systems (RIS) and National Innovation Systems (NIS). This section should explain the distinct features, theoretical frameworks, and interconnections between these two systems. 

 2. Clarify how the regional perspective relates to the national system in Ethiopia, and explicitly state whether the focus is on regional innovation within the broader national framework or purely national-level innovation policies. 

 3. Alternatively, if the primary focus is on the national system, the title and literature should be adjusted to reflect that. The author should replace the current references with relevant literature on national innovation systems and national development strategies. 

 4. If the intention is to maintain the focus on the regional level, the analysis should shift to regional innovation dynamics and regional development frameworks, ensuring alignment with the core concept of Regional Innovation Systems.

**2. Methodological Enhancement:**

 Incorporating a SWOT-TOWS analysis, as outlined in the article Samara, E., Kilintzis, P., Grammenou, A. *et al.* Towards Transformative Innovation Policy in Territorial Governance: Mapping the Relevance of RRI Elements. *J Knowledge Economy* (2024 [Ref-1]). https://doi.org/10.1007/s13132-024-02320-1, would provide a more structured and practical framework for developing improvement strategies. This methodology will help ensure that the recommendations are specific, actionable, and policy-oriented. Presenting the proposed strategies in a comprehensive table will also enhance the clarity and accessibility of the findings.

 By incorporating TOWS analysis, the author could: 

 1. Align internal strengths with external opportunities to propose proactive strategies. 

 2. Mitigate internal weaknesses by leveraging external opportunities or reducing threats. 

 3. Ensure a policy-focused approach with clear, tangible outputs that are relevant to stakeholders and decision-makers. 

**3. Policy Strategy Integration:**

 The suggested strategies should be consolidated into a coherent section or table that highlights key priorities and links them to the identified challenges and opportunities. This will create a stronger, policy-relevant narrative that aligns with international best practices in innovation system analysis.  

 These revisions will significantly improve the article’s impact, making it more relevant for both academic and policy audiences. Careful attention to these recommendations will enhance the paper’s conceptual consistency, methodological rigor, and practical relevance, ensuring its readiness for indexing.

Is the work clearly and accurately presented and does it cite the current literature?

Yes

If applicable, is the statistical analysis and its interpretation appropriate?

Not applicable

Are all the source data underlying the results available to ensure full reproducibility?

Yes

Is the study design appropriate and is the work technically sound?

Yes

Are the conclusions drawn adequately supported by the results?

Partly

Are sufficient details of methods and analysis provided to allow replication by others?

Yes

Reviewer Expertise:

Innovation systems, regional development, entrepreneurship

I confirm that I have read this submission and believe that I have an appropriate level of expertise to confirm that it is of an acceptable scientific standard, however I have significant reservations, as outlined above.

## References

[1] : Towards Transformative Innovation Policy in Territorial Governance: Mapping the Relevance of RRI Elements. *Journal of the Knowledge Economy* .2024; 10.1007/s13132-024-02320-1 10.1007/s13132-024-02320-1

# Response

**Published:** 2025-02-16

**Authors:** Kassahun Sube

**Affiliations:** Development studies, Addis Ababa University, Addis Ababa, Addis Ababa, Ethiopia

Dear Samara,

 I want to express my heartfelt gratitude for your invaluable insights and comments. Your feedback has not only presented a challenge but has also been incredibly helpful in enhancing my understanding of the subject matter.

 I took the time to review the article you recommended, and I found it to be extremely beneficial. It provided me with additional perspectives and knowledge that I greatly appreciate.

 Thank you once again for your support, and I look forward to applying what I've learned in our future discussions.

 Regards!

# Reviewer report: Reviewer response for version 1

**DOI:** 10.5256/f1000research.175551.r358945

**Published:** 2025-02-04

**Authors:** Shem Sikombe

**Affiliations:** 1 The Copperbelt University, Kitwe, Copperbelt Province, Zambia

**Title: Regional Innovation Systems as a Means for Development: Policies, Opportunities, and Challenges in Ethiopia**

**Introduction**

 Thank you for the opportunity to review and provide insights into this research. The study is highly relevant, given the increasing emphasis on Science, Technology, and Innovation (STI) as a driver of economic development.

 The article highlights the role of STI in fostering economic growth at both global and regional levels. Additionally, it provides a comprehensive review of policies supporting STI, thereby offering a well-defined contextual framework for the study.

 To enhance clarity, the research objectives should be explicitly stated. Based on the title and scope of the study, the following research objectives are suggested:

1. To review the policies supporting Regional Innovation Systems (RIS) in Ethiopia.
2. To explore the opportunities for leveraging RIS for economic development.
3. To identify the main challenges hindering RIS from contributing to economic development.

**Literature Review**

 The section titled "Science, Technology, and Innovation Policies (STIP) in Emerging Economies," which currently appears after the methods section, would be more appropriately positioned within the literature review. Integrating this section into the literature review will enhance the logical flow and coherence of the study.

**Methods**

 The methodology section needs further elaboration to ensure enhanced credibility. Specifically, the process of identifying and selecting documents for analysis needs clarification. The following aspects should be explicitly addressed:

1. What were the inclusion and exclusion criteria for document selection?
2. What specific topics or themes did these documents address?
3. Who authored these documents, and what credibility do they hold in the field?

The coding process and theme generation should be clearly explained. Furthermore, the approach to data saturation remains ambiguous. The study should clarify the following:

- How was data saturation determined?
- Were the themes inductively generated from the data, or were they deductively derived based on predefined categories?

**Findings**

 The findings section would benefit from a more structured thematic organisation. Grouping findings under specific themes such as challenges, opportunities, and policy implications would enhance clarity and readability. A possible thematic structure could be:

1. **Challenges in Promoting RIS**: A synthesis of the main challenges identified in the reviewed documents.
2. **Opportunities for RIS**: Potential avenues for leveraging RIS to drive economic growth.
3. **Policy Implications**: Key policy recommendations emerging from the study.

Overall, this research provides valuable insights into how RIS can support economic development in Ethiopia.

Is the work clearly and accurately presented and does it cite the current literature?

Yes

If applicable, is the statistical analysis and its interpretation appropriate?

Not applicable

Are all the source data underlying the results available to ensure full reproducibility?

No source data required

Is the study design appropriate and is the work technically sound?

Partly

Are the conclusions drawn adequately supported by the results?

Yes

Are sufficient details of methods and analysis provided to allow replication by others?

Partly

Reviewer Expertise:

Supply Chain Management, Research and Inovation, Knowledge Management, Operations Management and General Management.

I confirm that I have read this submission and believe that I have an appropriate level of expertise to confirm that it is of an acceptable scientific standard, however I have significant reservations, as outlined above.

# Response

**Published:** 2025-02-10

**Authors:** Kassahun Sube

**Affiliations:** Development studies, Addis Ababa University, Addis Ababa, Addis Ababa, Ethiopia

Dear Shem Sikombe,

 I appreciate your prompt response in reviewing our manuscript. My colleagues and I found your comments to be constructive, and we have accepted them. We are currently working to improve the manuscript based on your feedback and will provide an updated version once we receive comments from the other reviewers. Thank you for your time and expertise.

 Regards,

 Kassahun A. Sube,

 Corresponding Author
